# Synergistic binding sites in a hybrid ultramicroporous material for one-step ethylene purification from ternary C_2_ hydrocarbon mixtures

**DOI:** 10.1126/sciadv.abn9231

**Published:** 2022-06-08

**Authors:** Peixin Zhang, Yao Zhong, Yan Zhang, Zhenliang Zhu, Yuan Liu, Yun Su, Jingwen Chen, Shixia Chen, Zheling Zeng, Huabin Xing, Shuguang Deng, Jun Wang

**Affiliations:** 1 Chemistry and Chemical Engineering School, Nanchang University, Nanchang 330031, P.R. China.; 2 Key Laboratory of Biomass Chemical Engineering of Ministry of Education, College of Chemical and Biological Engineering, Zhejiang University, Hangzhou 310027, P.R. China.; 3 Jiangxi University of Chinese Medicine, Nanchang, 330031, Jiangxi, P.R. China.; 4 School for Engineering of Matter, Transport and Energy, Arizona State University, 551 E. Tyler Mall, Tempe, AZ 85287, USA.

## Abstract

One-step separation of C_2_H_4_ from ternary C_2_H_2_/C_2_H_4_/C_2_H_6_ hydrocarbon mixtures is of great significance in the industry but is challenging due to the similar sizes and physical properties of C_2_H_2_, C_2_H_4_, and C_2_H_6_. Here, we report an anion-pillared hybrid ultramicroporous material, CuTiF_6_-TPPY, that has the ability of selective recognition of C_2_H_4_ over C_2_H_2_ and C_2_H_6_. The 4,6-connected *fsc* framework of CuTiF_6_-TPPY exhibits semi–cage-like one-dimensional channels sustained by porphyrin rings and TiF_6_^2−^ pillars, which demonstrates the noticeably enhanced adsorption of C_2_H_2_ and C_2_H_6_ over C_2_H_4_. Dynamic breakthrough experiments confirm the direct and facile high-purity C_2_H_4_ (>99.9%) production from a ternary gas mixture of C_2_H_2_/C_2_H_6_/C_2_H_4_ (1/9/90, v/v/v) under ambient conditions. Computational studies and in situ infrared reveal that the porphyrin moieties with large π-surfaces form multiple van der Waals interactions with C_2_H_6_; meanwhile, the polar TiF_6_^2−^ pillars form C–H•••F hydrogen bonding with C_2_H_2_. In contrast, the recognition sites for C_2_H_4_ in the framework are less marked.

## Teaser

An anion-pillared HUM realizes one-step C_2_H_4_ purification from ternary C_2_ hydrocarbon mixture by synergistic binding sites.

## INTRODUCTION

As the largest feedstock in the petrochemical industry, the purification of olefins, e.g., ethylene and propylene, collectively accounts for approximately 0.3% of global energy (*1*, *2*). The annual production of ethylene (C_2_H_4_) exceeded 190 million metric tons in 2019 and will continuously expand in the foreseeable future (*3*). In industry, C_2_H_4_ is mainly produced by steam cracking of naphtha or thermal dehydrogenation of ethane, in which the impurities of acetylene (C_2_H_2_) and ethane (C_2_H_6_) are inevitably entrained (*4*). The trace amount of C_2_H_2_ (1000 to 5000 parts per million) will poison the catalysts of polyethylene production and even lead to an explosion; meanwhile, high levels of C_2_H_6_ will compromise the polymer production efficiency (*5*). Now, catalytic hydrogenation is used to remove C_2_H_2_ from C_2_H_4_ at high temperature and pressure (*6*), while the C_2_H_6_-C_2_H_4_ mixtures are separated by thermal-driven cryogenic distillation with large reflux ratio and >150 trays at −25°C and 23 bar (*7*). Therefore, physisorption-based separation with high-energy efficiency is considered as a promising alternative to separate C_2_H_4_ from C_2_H_2_-C_2_H_4_-C_2_H_6_ ternary mixtures in a single step under mild operation conditions (*8*, *9*).

Anion-pillared hybrid ultramicroporous materials (HUMs) are an intriguing subclass of metal-organic frameworks (MOFs); the pore size and chemistry can be exquisitely modulated through the reticular principle and crystal engineering strategies (*10*–*13*). Another important dimension is that the altering of anion pillars (e.g., SiF_6_^2−^, TiF_6_^2−^, GeF_6_^2−^, ZrF_6_^2−^, and NbOF_5_^2−^) can induce the distortion of pore shapes and the rotation of organic ligands (*14*, *15*), thus anion-pillared HUMs have emerged as the promising C_2_ light hydrocarbon adsorbents for binary separations such as C_2_H_2_/C_2_H_4_ (*16*), C_2_H_2_/CO_2_ (*17*), and C_2_H_4_/C_2_H_6_ (*18*). However, the single-step C_2_H_4_ purification from ternary C_2_ mixtures has never been achieved in adsorptive separations using anion-pillared HUMs, because the quadrupole moment and kinetic diameter of C_2_H_4_ [1.5 × 10^−26^ electrostatic unit (esu) cm^2^ and 4.1 Å] locate between those of C_2_H_2_ (7.2 × 10^−26^ esu cm^2^ and 3.3 Å) and C_2_H_6_ (0.65 × 10^−26^ esu cm^2^ and 4.4 Å) (*19*). Thus far, only nine reports have potentially realized the single-step separation of C_2_H_4_ from C_2_H_2_/C_2_H_4_/C_2_H_6_ ternary mixtures, and only seven MOFs have demonstrated their potentials in practical applications with dynamic breakthrough experiments (*5*, *20*–*27*).

The abundant polar anion pillars (MF_6_^2−^) in HUMs are strong C_2_H_2_ recognition sites as hydrogen bonding acceptors, enabling pervasive high C_2_H_2_ uptake and selectivity over C_2_H_4_ (*28*–*30*). For instance, Cu(4,4′-dipyridylsulfone)_2_(TiF_6_) (ZUL-100) exhibited the highest C_2_H_2_ adsorption capacity of 2.96 mmol g^−1^ at an ultralow pressure of 0.01 bar and resulted in an outstanding C_2_H_2_/C_2_H_4_ selectivity of 175 at 298 K and 1 bar (*31*). Regarding the C_2_H_4_/C_2_H_6_ separation, unsaturated C_2_H_4_ is preferentially adsorbed via the electron π-complexion mechanism, leading to the preferential C_2_H_4_ adsorption over C_2_H_6_ (*32*). In contrast, C_2_H_6_-selective adsorption can substantial reduce energy consumption and afford high-purity C_2_H_4_ by avoiding the C_2_H_4_ recovery through heating or vacuuming (*33*). To date, only limited low-polarity or aromatic-rich MOFs have exhibited such unusual adsorption behavior (*34*, *35*). Considering that C_2_H_6_ has larger polarizability (44.4 × 10^−25^ to 44.7 × 10^−25^ cm^3^ for C_2_H_6_ and 42.5 × 10^−25^ cm^3^ for C_2_H_4_) and van der Waals surface area (75 Å^2^ for C_2_H_6_ and 61 Å^2^ for C_2_H_4_), low-polarity MOFs featuring abundant aromatic or aliphatic ligands can yield selective C–H•••π and van der Waals interactions with C_2_H_6_ molecules (*25*). However, the organic ligands of existing anion-pillared HUMs fail to provide sufficient low-polarity π-surfaces to selectively bind C_2_H_6_ over C_2_H_4_. To the best of our knowledge, the selective adsorption of C_2_H_2_ and C_2_H_6_ over C_2_H_4_ from C_2_H_2_/C_2_H_4_/C_2_H_6_ ternary mixtures has never been documented in anion-pillared HUMs.

**Fig. 1.**
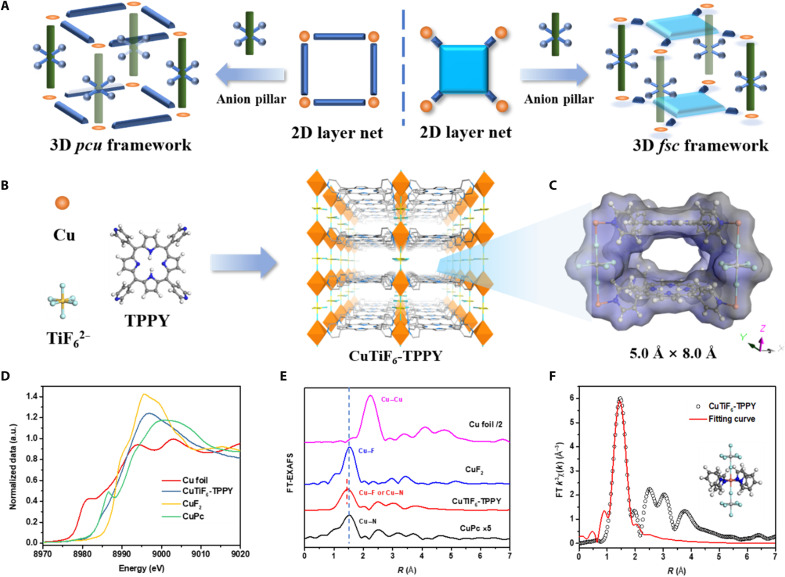
Schematic illustration of the structure of CuTiF_6_-TPPY. (**A**) Schematic illustration of the modularity with different 2D layer net and anion pillars that form 3D *pcu* or *fsc* topology anion-pillared HUMs. (**B**) Schematic illustration of the building blocks (Cu^II^, TiF_6_^2−^, and TPPY organic ligand) and the 3D *fsc* network topology of CuTiF_6_-TPPY. (**C**) Pore size and shape. (**D**) The normalized x-ray absorption near-edge structure (XANES) spectra at the Cu K-edge. a.u., arbitrary units. (**E**) Fourier transformation of the extended x-ray absorption fine structure (EXAFS) spectra in the *R* space. FT, Fourier transform. (**F**) The EXAFS fitting curve for CuTiF_6_-TPPY.

Here, we report an example of anion-pillared HUMs, CuTiF_6_-TPPY [5,10,15,20-tetra(4-pyridyl)-21h,23h-porphyrin], which can one-step separate C_2_H_4_ from C_2_H_2_/C_2_H_4_/C_2_H_6_ ternary mixtures. The rarely reported quadritopic pyridyl-based ligand creates semi–cage-like one-dimensional (1D) channels with a proper cavity size of 5.0 Å by 8.0 Å rather than the 1D penetrating frameworks using two connected linear ligands (Fig. 1A). The properly positioned anion pillars (TiF_6_^2−^) provide strong recognition sites for C_2_H_2_ with C–H•••F hydrogen bonds. Meanwhile, the porphyrin moieties with large π-surfaces form multiple van der Waals interactions (C–H•••N bonds) with C_2_H_6_. Those specific binding sites are absent for C_2_H_4_, which results in efficient single-step purification of C_2_H_4_ (purity, >99.9%) from a ternary mixture of C_2_H_2_/C_2_H_4_/C_2_H_6_ in one adsorption column at room temperature.

## RESULTS

### Pore structure and C_2_ adsorption property

The reaction of TPPY with Cu(BF_4_)_2_·4H_2_O and (NH_4_)_2_TiF_6_ in methanol solution at 60°C afforded a dark red powder of CuTiF_6_-TPPY (Fig. 1B; see the Supplementary Materials for synthetic and crystallographic details). Notably, CuTiF_6_-TPPY can also be successfully prepared by using other Cu salts such as Cu(NO_3_)_2_·3H_2_O and CuCl_2_·2H_2_O (fig. S5). Despite extensive attempts, high-quality single crystals of CuTiF_6_-TPPY cannot be obtained for single-crystal x-ray diffraction (XRD) studies. Therefore, multiple technologies were applied to determine the accurate crystal structure of CuTiF_6_-TPPY. First, the coordination environment of Cu ions on CuTiF_6_-TPPY was probed by Cu K-edge x-ray absorption fine structure (XAFS) analysis (*36*, *37*). The normalized x-ray absorption near-edge structure (XANES) curves of Cu K-edge of CuTiF_6_-TPPY located between Cu phthalocyanine (CuPc; Cu─N_4_) and Cu fluoride (CuF_2_; Cu─F_2_), indicating the hybrid coordination of Cu─N and Cu─F bonds (Fig. 1D). To confirm this result, the Fourier transform (FT) *k*^3^-weighted extended XAFS (EXAFS) spectrum of CuTiF_6_-TPPY showed a main peak at 1.52 Å (Fig. 1E), which has a small offset compared to that of CuPc (Cu─N bond; 1.53 Å) and CuF_2_ (Cu─F bond; 1.53 Å), also confirming the hybrid coordination environment. Notably, no Cu─Cu coordination peak was observed at 2.2 Å, which unambiguously implied the complete absence of Cu nanoparticles or clusters. The fitting of the Cu K-edge EXAFS spectrum indicated a total coordination number of 5.8 ± 0.8, constituting by an average number of 4 and 2 for Cu─N and Cu─F path, respectively (Fig. 1F and table S1). In addition, the wavelet transform (WT) plot of CuTiF_6_-TPPY with the maximum WT centered at 4.5 Å^−1^ (fig.S1), which is close to that of CuPc and CuF_2_ but far from that of the Cu─Cu bond (6 to 8 Å^−1^), also confirming the existence of the Cu─N and Cu─F bonds in CuTiF_6_-TPPY. Inductively coupled plasma optical emission spectroscopy (ICP-OES) reveals that the Cu and Ti contents on CuTiF_6_-TPPY are 9.07 weight % (wt %) and 7.06 wt %, respectively, which are close to the theoretical contents of Cu (7.64 wt %) and Ti (5.73 wt %). The calculated molar ratio of Cu and Ti is close to 1:1, which confirms the absence of Cu chelation on porphyrin rings. Combined with the C and N contents by ultimate elemental analysis (table S3), the unit formula of CuTiF_6_-TPPY was determined to be Cu(TPPY)(TiF_6_). The x-ray photoelectron spectroscopy measurement also confirmed the element compositions in CuTiF_6_-TPPY (fig. S6 and table S3).

The Rietveld refinement of powder XRD (PXRD) data revealed that the as-synthesized CuTiF_6_-TPPY crystallizes in the orthorhombic crystal system with cell parameters of *a* = 13.858, *b* = 13.788, and *c* = 8.232 (fig. S2 and table S2) (*38*). The simulated PXRD pattern matches well with the experimental one, confirming the high phase purity of as-synthesized CuTiF_6_-TPPY (fig. S3). Individually, each Cu(II) atom was connected by four pyridyl groups from independent TPPY ligands to form a 2D layer network, which was further pillared by TiF_6_^2−^ anions and thus affords a 3D framework without interpenetration (Fig. 1B and fig. S7). The TiF_6_^2−^ pillars and pyridine rings are interconnected through hydrogen bonds with C–H•••F distances of 2.27 and 2.29 Å, leading to the titling of pyridine rings by 69.4° with respect to the crystal axials (fig. S8). Because of the high symmetries of the four connected TPPY linker and coordination model, CuTiF_6_-TPPY exhibits one type of semi–cage-like 1D channels with a cavity size of 5.0 Å by 8.0 Å (Fig. 1C and fig. S9), which is smaller than the 1D penetrating frameworks using two connecting linear ligand of SiFSiX-1-Cu (~8.0 Å; 1 = 4, 4′-bipyridine; fig. S10) (*15*, *28*). Thermogravimetric analysis showed that the guest molecules can be completely removed at 80°C, and CuTiF_6_-TPPY showed a good thermostability until 320°C (fig. S11).

**Fig. 2.**
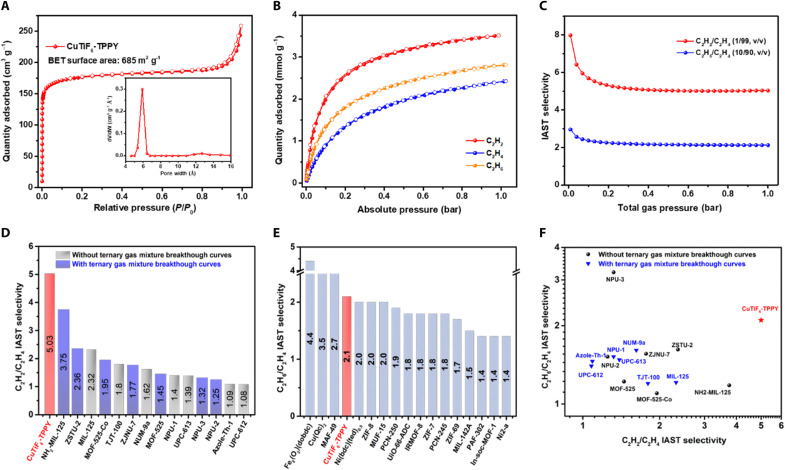
Single-component gas adsorption properties. (**A**) N_2_ adsorption-desorption isotherm at 77 K and NLDFT PSD curve. (**B**) C_2_H_2_, C_2_H_4_, and C_2_H_6_ adsorption isotherms at 298 K. (**C**) C_2_H_2_/C_2_H_4_ and C_2_H_6_/C_2_H_4_ ideal adsorbed solution theory (IAST) selectivity. Comparison plot of (**D**) C_2_H_2_/C_2_H_4_ IAST selectivity, (**E**) C_2_H_6_/C_2_H_4_ IAST selectivity, and (**F**) comprehensive C_2_ separation performances with representative porous materials.

Before adsorption measurements, CuTiF_6_-TPPY was exchanged with methanol and acetone and activated at 333 K under a high vacuum (<5 μm of Hg) for 12 hours. The activated CuTiF_6_-TPPY exhibited the intact PXRD pattern, implying the indistinctive structure distortion and rigid frameworks (fig. S3). The permanent porosity of CuTiF_6_-TPPY was investigated by N_2_ adsorption-desorption isotherm at 77 K (Fig. 2A), and the typical type I adsorption isotherm indicates the microporous nature of CuTiF_6_-TPPY (*39*). The Brunauer-Emmett-Teller (BET)–specific surface area and the pore volume were determined to be 685 m^2^ g^−1^ and 0.32 cm^3^ g^−1^, respectively (Fig. 2A and fig. S12). The pore size distribution (PSD) that was determined by the nonlocal density functional theory (NLDFT) method centered at 6.0 Å agreed well with the pore size (5.0 Å by 8.0 Å) derived from the crystal analysis. Notably, the pore structures can be remained after repeated breakthrough cycles and 4 months of storage, suggesting excellent structural robustness (fig. S13). Meanwhile, CuTiF_6_-TPPY can maintain its crystalline after soaking in various organic solvents for 1 week as verified by PXRD patterns (fig. S4).

To explore the adsorption and separation performances of CuTiF_6_-TPPY, single-component adsorption isotherms of C_2_ hydrocarbons were measured at 273, 288, and 298 K (Fig. 2B and fig. S14). Specifically, CuTiF_6_-TPPY exhibited an unusual adsorption behavior in the order of C_2_H_2_ > C_2_H_6_ > C_2_H_4_, indicating the potential of one-step separation C_2_H_4_ from C_2_H_2_/C_2_H_4_/C_2_H_6_ ternary mixtures. At 298 K and 1 bar, the uptake amount of C_2_H_2_, C_2_H_6_, and C_2_H_4_ on CuTiF_6_-TPPY reaches 3.62, 2.82, and 2.42 mmol g^−1^, giving a C_2_H_2_/C_2_H_4_ and C_2_H_6_/C_2_H_4_ uptake ratio of 1.50 and 1.17, respectively, outperforming most reported MOFs (table S7). In addition, the adsorption isotherms of C_2_ hydrocarbons are reversible, implying that CuTiF_6_-TPPY can be easily regenerated.

Furthermore, the ideal adsorbed solution theory (IAST) was applied to evaluate the C_2_H_2_/C_2_H_4_ and C_2_H_6_/C_2_H_4_ selectivity. Figure 2C showed that the IAST selectivity of C_2_H_2_/C_2_H_4_ (1/99) and C_2_H_6_/C_2_H_4_ (10/90) reaches 5.03 and 2.12 on CuTiF_6_-TPPY at 298 K and 1.0 bar. Note that the C_2_H_2_/C_2_H_4_ (1/99) selectivity (5.03) is the highest among all seven MOFs that achieved one-step C_2_H_4_ separation from C_2_H_2_/C_2_H_4_/C_2_H_6_ ternary mixtures (Fig. 2D). Meanwhile, the IAST selectivity of C_2_H_6_/C_2_H_4_ (10/90) is also superior to most top-ranking C_2_H_6_-selective MOFs (Fig. 2E). Therefore, CuTiF_6_-TPPY can be considered as the benchmark for one-step C_2_H_4_ separation from C_2_H_2_/C_2_H_4_/C_2_H_6_ ternary mixtures concerning the simultaneously outstanding C_2_H_2_/C_2_H_4_ and C_2_H_6_/C_2_H_4_ selectivities (Fig. 2F). Similarly, CuTiF_6_-TPPY also exhibited high IAST selectivities of 5.47 and 2.12 on C_2_H_2_/C_2_H_4_ (50/50) and C_2_H_6_/C_2_H_4_ (50/50) binary gas mixtures (fig. S18). Compared to the analogous SIFSIX-1-Cu, the C_2_H_6_/C_2_H_4_ separation performance was significantly improved attributing to the porphyrin moieties with large π-surfaces of CuTiF_6_-TPPY (fig. S15).

To evaluate the affinity of CuTiF_6_-TPPY to guest adsorbates, the isosteric heat of adsorption (*Q*_st_), a quantitative assessment of binding affinity, was calculated on the basis of fitted isotherms at three different temperatures by the Clausius-Clapeyron equation (*40*). The isotherm fitting details are provided in the Supplementary Materials (table S5 and fig. S17). The *Q*_st_ of C_2_H_2_ and C_2_H_6_ is calculated to be 36.5 and 34.2 kJ mol^−1^ at zero coverage on CuTiF_6_-TPPY (fig. S19), both higher than that of C_2_H_4_ (29.6 kJ mol^−1^). This result indicates that CuTiF_6_-TPPY has higher affinities toward C_2_H_2_ and C_2_H_6_ than C_2_H_4_, which is consistent with the order of adsorption capacities. All the *Q*_st_ of C_2_ adsorbates are located in the physisorption range and lower than many ultramicroporous MOFs (table S6), implying the low energy consumption for regeneration (*41*).

### Dynamic breakthrough experiments

**Fig. 3.**
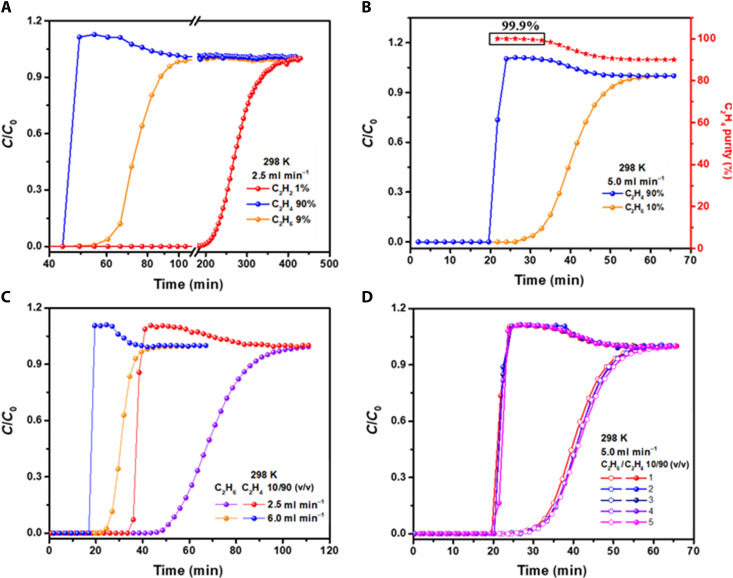
Dynamic breakthrough curves and cycling tests. The dynamic breakthrough curve of (**A**) C_2_H_2_/C_2_H_6_/C_2_H_4_ (1/9/90, v/v/v) mixture at 2.5 ml/min. Dynamic breakthrough curves of C_2_H_6_/C_2_H_4_ (10/90, v/v) binary mixture (**B**) at the flow rate of 5.0 ml/min and (**C**) 2.5 and 6.0 ml/min. (**D**) Five continuous breakthrough cycles at the flow rate of 5.0 ml/min on CuTiF_6_-TPPY at 298 K.

Dynamic transient breakthrough experiments were further carried out to evaluate the actual performances of CuTiF_6_-TPPY for separating C_2_H_2_/C_2_H_6_/C_2_H_4_ (1/9/90, v/v/v) ternary mixture at a flow rate of 2.5 ml min^−1^ under ambient conditions. As shown in Fig. 3A, C_2_H_4_ first eluted through the column at 49.5 min, followed by C_2_H_6_ at 60.5 min; whereas, the breakthrough of C_2_H_2_ occurred at an elongated time of 220 min. Note that the ability to remove trace C_2_H_2_ is the best among all the one-step C_2_H_4_ purification adsorbents. The concentration of C_2_H_4_ at the outlet was monitored by online gas chromatography, and high-purity C_2_H_4_ (>99.9%) can be obtained in one step with a collection window of 11 min. In addition, the clean separation of C_2_H_4_ can also be obtained at a higher flow rate of 5.0 ml min^−1^ (fig. S21). In contrast, the analogous SIFSIX-1-Cu can barely separate binary C_2_H_6_/C_2_H_4_ (10/90, v/v) and ternary C_2_H_2_/C_2_H_6_/C_2_H_4_ (1/9/90, v/v/v) gas mixtures (fig. S16).

Considering the close breakthrough time of C_2_H_6_ and C_2_H_4_ compared to that of C_2_H_2_/C_2_H_4_, detailed breakthrough experiments with various C_2_H_6_/C_2_H_4_ compositions and flow rates were conducted. As shown in Fig. 3B, for C_2_H_6_/C_2_H_4_ (10/90, v/v) binary gas mixture, C_2_H_4_ and C_2_H_6_ eluted through the column at 19.6 and 28.4 min, respectively, affording a time interval of 8.8 min to produce high-purity C_2_H_4_ (>99.9%). The productivity of C_2_H_4_ (>99.9%) was calculated to be 1.09 mmol g^−1^, which is superior or comparable to top-performing materials, such as Azote-Th-1 (1.13 mmol g^−1^; 10/90, v/v) (*21*), Zn(batz) (MAF-49) (0.28 mmol g^−1^; 50/50, v/v) (*42*), Fe_2_(O_2_)(dobdc) (0.79 mmol g^−1^; 50/50, v/v) (*33*), and Mn_5_^II^Mn^III^(u_3_-O)_2_-(CH_3_COO)_3_(Tripp)_2_(BDC)_3_ (NPU-1) (0.28 mmol g^−1^; 1/2, v/v) (*20*). Moreover, clean separations can also be obtained at total flow rates of 2.5 and 6.0 ml min^−1^ (Fig. 2C). Similarly, the breakthrough curve of C_2_H_6_/C_2_H_4_ (50/50, v/v) revealed that the high-purity C_2_H_4_ (99.9%) can be collected from a relatively low concentration of C_2_H_4_ at a large flow rate of 8.0 ml min^−1^ (fig. S21). For practical applications, the material stability is critical; five continuous cycles for the separation of C_2_H_6_/C_2_H_4_ mixture after facile regeneration with a He flow of 20 ml min^−1^ at 298 K displayed no noticeable deterioration in retention time during the stability test (Fig. 3D and fig. S23). In regeneration processes, high-purity C_2_H_6_ cannot be obtained even at smaller He flow rates of 5 and 10 ml min^−1^ (fig. S24). Time-dependent gas uptake profiles were further gravimetrically recorded (fig. S25A), almost identical slopes of C_2_H_4_ and C_2_H_6_ for each pressure stage confirmed the close adsorption and desorption rates. Furthermore, in the desorption curves (fig. S25B), rapid gas desorptions were both completed within 3 min due to their close interaction strengths with CuTiF_6_-TPPY. Note that this phenomenon does not compromise the one-step separation performances because C_2_H_4_ is directly collected at the exit of adsorption column rather than in the desorption process. Moreover, the reproducibility for the synthesis process and separation performances of CuTiF_6_-TPPY were demonstrated by the intact XRD patterns, C_2_ adsorption capacities, and dynamic breakthrough performances on 10 parallelly synthesized batches (figs. S30 to S32).

### Computational simulation studies

**Fig. 4.**
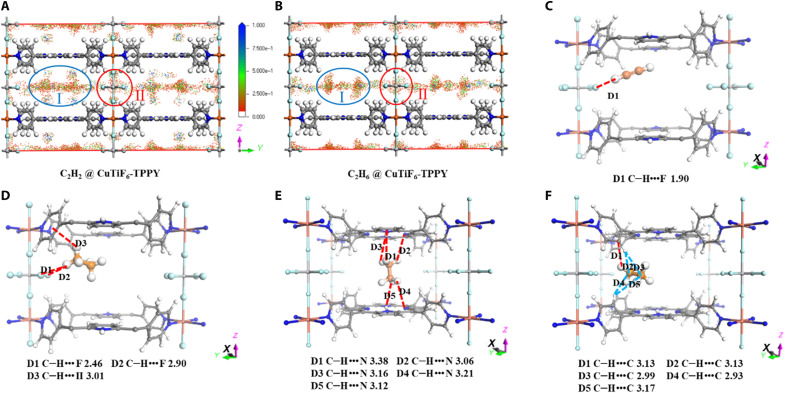
Theoretical studies for C_2_ distribution density and binding sites in CuTiF_6_-TPPY. Computational simulations for the density distribution of (**A**) C_2_H_2_ and (**B**) C_2_H_6_ on CuTiF_6_-TPPY at 100 kPa and 298 K. The DFT calculated binding sites of (**C**) C_2_H_2_ and (**D** to **F**) C_2_H_6_ in CuTiF_6_-TPPY. The closest contacts between framework atoms and the gas molecules are defined by the distances (in angstrom), and the distances include the van der Waals radius of atoms. [Framework: C, gray (80%); H, white; N, blue; F, cyan; Cu, pink; Ti, silvery; gas: C, orange; H, white].

To reveal the host-guest interactions between the framework and adsorbates, we performed the grand canonical Monte Carlo (GCMC) calculations, first-principles dispersion-corrected DFT calculations, and in situ infrared (IR) experiments. The C_2_ gases were adsorbed in two major areas, i.e., the organic ligand of the TPPY region (region I) and the TiF_6_^2−^ anion-pillared region (region II; Fig. 4, A and B). The contribution density for C_2_ gases is in the order of C_2_H_2_ > C_2_H_6_ > C_2_H_4_ that is consistent with the adsorption capacities (fig. S27). As for region II, the contribution densities for C_2_H_2_ and C_2_H_6_ were higher than that of C_2_H_4_, confirming that the TiF_6_^2−^ anions could provide stronger interactions with C_2_H_2_ and C_2_H_6_ over C_2_H_4_ (Fig. 4, A and B, and fig. S26A). As for region I, the C_2_H_2_ molecules were uniformly distributed along with the whole porphyrin ring; C_2_H_6_ molecules were mainly adsorbed at the center of porphyrin rings and the junction of pyridine and porphyrin rings. In contrast, C_2_H_4_ molecules were loosely distributed near the porphyrin rings (fig. S27B). Meanwhile, DFT calculations were conducted to identify the adsorption sites for C_2_ gases. The C_2_H_2_ molecule is adsorbed around TiF_6_^2−^ anions via a strong H bonding (C–H•••F 1.90 Å), yielding C_2_H_2_ binding energy of −44.1 kJ mol^−1^ (Fig. 4C). Meanwhile, the C_2_H_6_ molecules can be captured by three adsorption sites that are provided by both TiF_6_^2−^ anion and TPPY ligand: (i) the synergy of C–H•••π (3.01 Å) and C–H•••F (2.46 Å and 2.90 Å) interactions (Fig. 4D), (ii) the C–H•••N interactions with porphyrin rings (Fig. 4E), and (iii) multiple van der Waals forces between C_2_H_6_ and pyridine rings (Fig. 4f). The C_2_H_6_ binding energy at three sites is calculated to be −40.75, −36.2, and −40.2 kJ mol^−1^, respectively. In sharp contrast, the C_2_H_4_ molecules are weakly adsorbed by the C–H•••F bonding with TiF_6_^2−^ anion and multiple C–H•••N van der Waals interactions between two interlayers of TPPY ligands with much longer corresponding bonding distances (fig. S26). The C_2_H_4_ binding energy is calculated to be −28.4 and −25.0 kJ mol^−1^ at two binding sites, respectively. The trend and value of calculated binding energy between CuTiF_6_-TPPY and C_2_ adsorbates are similar to the experimental adsorption heat at zero coverage, confirming that anion pillars (TiF_6_^2−^) with strong polarization and porphyrin moieties with large π-surfaces played a synergistic role in the confined micropore channels for the selective recognition of C_2_H_2_ and C_2_H_6_ over C_2_H_4_. Furthermore, the in situ IR experiments with corresponding C_2_ gas loadings confirmed the relatively strong interactions between C_2_H_2_/C_2_H_6_ and CuTiF_6_-TPPY (fig. S28 and detailed discussion in the Supplementary Materials). The time-dependent in situ IR spectra with C_2_ gas loadings indicated that C_2_H_2_ and C_2_H_6_ can be adsorbed in a stronger and faster manner than C_2_H_4_ by CuTiF_6_-TPPY (fig. S29).

## DISCUSSION

In summary, we first synthesized and reported an anion-pillared HUM, CuTiF_6_-TPPY, that can one-step separate C_2_H_4_ from ternary C_2_H_2_/C_2_H_4_/C_2_H_6_ gas mixtures. The low-polarity π-surface areas provided by the porphyrin rings of TPPY can selectively adsorb C_2_H_6_ molecules via multiple van der Waals interactions. Meanwhile, the abundant TiF_6_^2−^ anions can strongly capture C_2_H_2_. As a result, CuTiF_6_-TPPY exhibited a higher adsorption capacity of C_2_H_2_ (3.62 mmol g^−1^) and C_2_H_6_ (2.82 mmol g^−1^) than that of C_2_H_4_ (2.42 mmol g^−1^) at 298 K and 1.0 bar. Notably, the highest C_2_H_2_/C_2_H_4_ IAST selectivity of 5.03 was obtained among the seven MOFs with the same adsorption behaviors. The superb IAST selectivity of C_2_H_6_/C_2_H_4_ (2.12) also outperformed most top-ranking MOFs. Dynamic breakthrough experiments with ternary and binary C_2_ gas mixtures have confirmed the direct and facile production of high-purity C_2_H_4_ (99.9%). Moreover, DFT calculations demonstrated the synergistic recognition sites by both polar anion pillars and porphyrin rings with large π-surface areas.

## MATERIALS AND METHODS

### Chemicals

All reagents were analytical grade and used as received without further purification. Cu(BF_4_)_2_·4H_2_O, Cu(NO_3_)_2_·3H_2_O, CuCl_2_·2H_2_O, and (NH_4_)_2_TiF_6_ were purchased from Aladdin Reagent Co. Ltd. TPPY was purchased from Jilin Province Extension Technology Co. Ltd. Ultrahigh purity–grade He (99.999%), N_2_ (99.999%), C_2_H_2_ (99.9%), C_2_H_4_ (99.99%), C_2_H_6_ (99.99%), and mixed gas (C_2_H_6_/C_2_H_4_ = 10/90, v/v; C_2_H_6_/C_2_H_4_/He = 25/25/50, v/v/v; C_2_H_2_/C_2_H_6_/C_2_H_4_ = 1/90/9, v/v/v) were purchased from Nanchang Jiangzu gas Co. Ltd. (China) and used for all measurements.

### Preparation of powder CuTiF_6_-TPPY

A preheated water solution (2 ml) of Cu(BF_4_)_2_·4H_2_O (0.2 mmol) and (NH_4_)_2_TiF_6_ (0.2 mmol) were dropped into a preheated methanol solution (30 ml) of TPPY (0.1 mmol). Then, the mixture was heated at 333 K for 24 hours. The obtained dark red powder was exchanged with methanol and acetone for a day, respectively. CuTiF_6_-TPPY can also be prepared by using other Cu salts such as Cu(NO_3_)_2_·3H_2_O and CuCl_2_·2H_2_O at the same condition.

### Sample characterization

PXRD patterns were collected using a PANalytical Empyrean Series 2 diffractometer with Cu─Ka radiation, at room temperature, with a step size of 0.0167°, a scan time of 15 s per step, and 2θ ranging from 5° to 50°. The contents of Cu and Ti were measured by ICP-OES (Agilent 5110, USA). The ultimate element analysis was conducted using a CHNS elemental analyzer (Vario MICRO). The N_2_ adsorption-desorption isotherms at 77 K were measured on a Micromeritics ASAP 2460 volumetric adsorption apparatus. The apparent BET-specific surface area was calculated using the adsorption branch with the relative pressure *P*/*P*_0_ in the range of 0.005 to 0.3. The total pore volume (*V*_tot_) was calculated on the basis of the adsorbed amount of nitrogen at the *P*/*P*_0_ of 0.99. The PSD was calculated using the NLDFT methodology with nitrogen adsorption isotherm data and assuming a slit pore model. The time-dependent gas uptake profiles were recorded on an Intelligent Gravimetric Analyzer (IGA-100, HIDEN). The pressure was raised at a rate of 100 mbar min^−1^ and kept for 60 min to reach full adsorption equilibriums.

### Gas adsorption measurements

The C_2_H_2_, C_2_H_4_, and C_2_H_6_ adsorption-desorption isotherms at 273, 288, and 298 K were measured volumetrically by the Micromeritics ASAP 2460 adsorption apparatus for pressures up to 1.0 bar. Before adsorption measurements, the samples were degassed using a high vacuum pump (<5 μm of Hg) at 333 K for over 12 hours.

### Breakthrough experiments

The dynamic breakthrough experiments were carried out in a homemade apparatus under ambient conditions. The samples were activated under vacuum at 100°C for 12 hours and loaded (1.8 or 1.3 g) to the adsorption bed (Φ 6 mm by 150 mm). A carrier gas (He of ≥99.999%) was used to purge the adsorption bed for 1 hour, and then the gas flow was switched to the desired gas mixture at a certain flow rate. The recovery gas was connected to an analyzer port coupled with gas chromatography (7890B, Agilent) with a flame ionization detector.

### In situ IR spectroscopic measurements

All the IR spectroscopic data are recorded in a Nicolet 6700 FTIR spectrometer (Thermo Fisher Scientific Inc., USA) equipped with a liquid N_2_-cooled mercury cadmium telluride MCT-A detector. A vacuum cell, purchased from Specac Ltd., UK (product number P/N 5850c), is placed in the sample compartment of the IR spectrometer with the sample at the focal point of the beam. The cell is connected to different gas lines (C_2_H_2_, C_2_H_4_, and C_2_H_6_) and a vacuum line for evacuation. The MOF sample (powder, ∼30 mg) was placed in the cell and first annealed at 80°C under vacuum for activation and then cooled to room temperature for recording the reference spectrum and subsequent loading gas. C_2_H_2_ was introduced into the cell, and the spectra were recorded during the gas exposure until 50 min. After fully evacuating the same sample by pumping the cell, the reference spectrum was taken again, loading of C_2_H_4_ and C_2_H_6_ was performed separately, and the IR data were recorded in the same manner.

### DFT calculations

First-principles DFT calculations were performed using the Materials Studio’s CASTEP code. All calculations were conducted under the generalized gradient approximation with Perdew-Burke-Ernzerhof. A semiempirical addition of dispersive forces to conventional DFT was included in the calculation to account for van der Waals interactions. A cutoff energy of 544 eV and a 2 × 2 × 2 *k*-point mesh were found to be enough for the total energy coverage within 0.01 meV atom^−1^. The structures of the synthesized materials were first optimized from the reported crystal structures. To obtain the binding energy, the pristine structure and an isolated gas molecule placed in a supercell (with the same cell dimensions as the pristine crystal structure) were optimized and relaxed as references. C_2_H_2_, C_2_H_4_, and C_2_H_6_ gas molecules were then introduced to different locations of the channel pore, followed by a full structural relaxation. The static binding energy was calculated by the equation *E*_B_ = *E*(gas) + *E*(adsorbent) − *E*(adsorbent + gas).

### GCMC calculations

All the GCMC simulations were performed in MS 2017R2 package. The crystal structure of the CuTiF_6_-TPPY was chosen after the DFT geometry optimization. The framework and the individual C_2_H_2_, C_2_H_4_, and C_2_H_6_ were considered to be rigid during the simulation. The charges for atoms of the CuTiF_6_-TPPY and C_2_ gases were derived from the Mulliken method. The simulations adopted the fixed pressure task, the Metropolis method in sorption module, and the universal force field. The interaction energies between the adsorbed molecules and the framework were computed through the Coulomb and Lennard-Jones 6-12 (LJ) potentials. The cutoff radius chosen was 18.5 Å for the LJ potential, and the electrostatic interactions were handled using the Ewald summation method. The loading steps and the equilibration steps were 1 × 10^7^, and the production steps were 1 × 10^7^.

### X-ray absorption spectroscopy

The x-ray absorption spectroscopy measurements at Cu K (*E*_0_ = 8983 eV) edge were collected on the beamline BL01C1 in the National Synchrotron Radiation Research Center. The energy was calibrated accordingly to the absorption edge of pure Cu foil. The radiation was monochromatized by a Si (111) double-crystal monochromator. XANES and EXAFS data reduction and analysis were processed by Athena software (version 0.9.26) for background, pre-edge line, and post-edge line calibrations. The chemical valence of Cu in the samples was determined by the comparison with the reference Cu foil, CuF_2_, and CuPc. For the EXAFS part, the FT data in *R* space were analyzed by applying the first-shell approximate model for Cu─N contribution. The passive electron factor S0 was determined by fitting the experimental data on Cu foil and then fixed for future analysis of the measured samples.

## Supplementary Materials

### This PDF file includes:

Figs. S1 to S32 Tables S1 to S7 References

## References

[1] D. S. Sholl, R. P. Lively, Seven chemical separations to change the world. Nature 532, 435–437 (2016). 27121824 10.1038/532435a

[2] J. Y. Lin, Molecular sieves for gas separation. Science 353, 121–122 (2016). 27387937 10.1126/science.aag2267

[3] I. Amghizar, L. A. Vandewalle, K. M. Van Geem, G. B. Marin, New trends in olefin production. Engineering 3, 171–178 (2017).

[4] T. Ren, M. Patel, K. Blok, Olefins from conventional and heavy feedstocks: Energy use in steam cracking and alternative processes. Energy 31, 425–451 (2006).

[5] H. G. Hao, Y. F. Zhao, D. M. Chen, J. M. Yu, K. Tan, S. Ma, Y. Chabal, Z. M. Zhang, J. M. Dou, Z. H. Xiao, Simultaneous trapping of C_2_H_2_ and C_2_H_6_ from a ternary mixture of C_2_H_2_/C_2_H_4_/C_2_H_6_ in a robust metal–Organic framework for the purification of C_2_H_4_. Angew. Chem. Int. Ed. 130, 16299–16303 (2018). 10.1002/anie.201809884 30338921

[6] F. Studt, F. Abild-Pedersen, T. Bligaard, R. Z. Sørensen, C. H. Christensen, J. K. Nørskov, Identification of non-precious metal alloy catalysts for selective hydrogenation of acetylene. Science 320, 1320–1322 (2008). 18535238 10.1126/science.1156660

[7] A. Cadiau, K. Adil, P. Bhatt, Y. Belmabkhout, M. Eddaoudi, A metal-organic framework–based splitter for separating propylene from propane. Science 353, 137–140 (2016). 27387945 10.1126/science.aaf6323

[8] H. Li, L. Li, R.-B. Lin, W. Zhou, Z. Zhang, S. Xiang, B. Chen, Porous metal-organic frameworks for gas storage and separation: Status and challenges. EnergyChem 1, 100006 (2019). 10.1016/j.enchem.2019.100006 PMC11071076 38711814

[9] Y. Yang, L. Li, R.-B. Lin, Y. Ye, Z. Yao, L. Yang, F. Xiang, S. Chen, Z. Zhang, S. Xiang, Ethylene/ethane separation in a stable hydrogen-bonded organic framework through a gating mechanism. Nat. Chem. 13, 933–939 (2021). 34239085 10.1038/s41557-021-00740-z

[10] O. M. Yaghi, M. J. Kalmutzki, C. S. Diercks, *Introduction to Reticular Chemistry: Metal-Organic Frameworks and Covalent Organic Frameworks* (Wiley, 2019).

[11] O. M. Yaghi, M. O’Keeffe, N. W. Ockwig, H. K. Chae, M. Eddaoudi, J. Kim, Reticular synthesis and the design of new materials. Nature 423, 705–714 (2003). 12802325 10.1038/nature01650

[12] L. Yang, S. Qian, X. Wang, X. Cui, B. Chen, H. Xing, Energy-efficient separation alternatives: Metal–organic frameworks and membranes for hydrocarbon separation. Chem. Soc. Rev. 49, 5359–5406 (2020). 32579620 10.1039/c9cs00756c

[13] R.-B. Lin, Z. Zhang, B. Chen, Achieving high performance metal-organic framework materials through pore engineering. Acc. Chem. Res. 54, 3362–3376 (2021). 34399577 10.1021/acs.accounts.1c00328

[14] L. Yang, X. Cui, Q. Yang, S. Qian, H. Wu, Z. Bao, Z. Zhang, Q. Ren, W. Zhou, B. Chen, A single-molecule propyne trap: Highly efficient removal of propyne from propylene with anion-pillared ultramicroporous materials. Adv. Mater. 30, 1705374 (2018). 10.1002/adma.201705374 29345384

[15] L. Li, H. M. Wen, C. He, R. B. Lin, R. Krishna, H. Wu, W. Zhou, J. Li, B. Li, B. Chen, A metal-organic framework with suitable pore size and specific functional sites for the removal of trace propyne from propylene. Angew. Chem. Int. Ed. 130, 15403–15408 (2018). 10.1002/anie.201809869 30240522

[16] L. Yang, A. Jin, L. Ge, X. Cui, H. Xing, A novel interpenetrated anion-pillared porous material with high water tolerance afforded efficient C_2_H_2_/C_2_H_4_ separation. Chem. Comm. 55, 5001–5004 (2019). 30968882 10.1039/c9cc00976k

[17] M. Jiang, X. Cui, L. Yang, Q. Yang, Z. Zhang, Y. Yang, H. Xing, A thermostable anion-pillared metal-organic framework for C_2_H_2_/C_2_H_4_ and C_2_H_2_/CO_2_ separations. Chem. Eng. J. 352, 803–810 (2018).

[18] Z. Zhang, Q. Ding, X. Cui, X.-M. Jiang, H. Xing, Fine-tuning and selective-binding within an anion-functionalized ultramicroporous metal-organic framework for efficient olefin/paraffin separation. ACS Appl. Mater. Interfaces 12, 40229–40235 (2020). 32805845 10.1021/acsami.0c07800

[19] J.-R. Li, R. J. Kuppler, H.-C. Zhou, Selective gas adsorption and separation in metal-organic frameworks. Chem. Soc. Rev. 38, 1477–1504 (2009). 19384449 10.1039/b802426j

[20] B. Zhu, J.-W. Cao, S. Mukherjee, T. Pham, T. Zhang, T. Wang, X. Jiang, K. A. Forrest, M. J. Zaworotko, K.-J. Chen, Pore engineering for one-step ethylene purification from a three-component hydrocarbon mixture. J. Am. Chem. Soc. 143, 1485–1492 (2021). 33439004 10.1021/jacs.0c11247 PMC8297724

[21] Z. Xu, X. Xiong, J. Xiong, R. Krishna, L. Li, Y. Fan, F. Luo, B. Chen, A robust Th-azole framework for highly efficient purification of C_2_H_4_ from a C_2_H_4_/C_2_H_2_/C_2_H_6_ mixture. Nat. Commun. 11, 1–9 (2020). 32572030 10.1038/s41467-020-16960-9 PMC7308359

[22] Y. Wang, C. Hao, W. Fan, M. Fu, X. Wang, Z. Wang, L. Zhu, Y. Li, X. Lu, F. Dai, Z. Kang, R. Wang, W. Guo, S. Hu, D. Sun, One-step ethylene purification from an acetylene/ethylene/ethane ternary mixture by cyclopentadiene cobalt-functionalized metal–organic frameworks. Angew. Chem. Int. Ed. 60, 11350–11358 (2021). 10.1002/anie.202100782 33661542

[23] S.-Q. Yang, F.-Z. Sun, P. Liu, L. Li, R. Krishna, Y.-H. Zhang, Q. Li, L. Zhou, T.-L. Hu, Efficient purification of ethylene from C_2_ hydrocarbons with an C_2_H_6_/C_2_H_2_-selective metal–organic framework. ACS Appl. Mater. Interfaces 13, 962–969 (2020). 33370532 10.1021/acsami.0c20000

[24] L. Fan, P. Zhou, X. Wang, L. Yue, L. Li, Y. He, Rational construction and performance regulation of an In(III)–tetraisophthalate framework for one-step adsorption-phase purification of C_2_H_4_ from C_2_ hydrocarbons. Inorg. Chem. 60, 10819–10829 (2021). 34197707 10.1021/acs.inorgchem.1c01560

[25] Z. Jiang, L. Fan, P. Zhou, T. Xu, S. Hu, J. Chen, D.-L. Chen, Y. He, An aromatic-rich cage-based MOF with inorganic chloride ions decorating the pore surface displaying the preferential adsorption of C_2_H_2_ and C_2_H_6_ over C_2_H_4_. Inorg. Chem. Front. 8, 1243–1252 (2021).

[26] R. E. Sikma, N. Katyal, S.-K. Lee, J. W. Fryer, C. G. Romero, S. K. Emslie, E. L. Taylor, V. M. Lynch, J.-S. Chang, G. Henkelman, Low-valent metal ions as MOF pillars: A new route toward stable and multifunctional MOFs. J. Am. Chem. Soc. 143, 13710–13720 (2021). 34410114 10.1021/jacs.1c05564

[27] P. Liu, Y. Wang, Y. Chen, J. Yang, X. Wang, L. Li, J. Li, Construction of saturated coordination titanium-based metal–organic framework for one-step C_2_H_2_/C_2_H_6_/C_2_H_4_ separation. Sep. Purif. Technol. 276, 119284 (2021).

[28] X. Cui, K. Chen, H. Xing, Q. Yang, R. Krishna, Z. Bao, H. Wu, W. Zhou, X. Dong, Y. Han, Pore chemistry and size control in hybrid porous materials for acetylene capture from ethylene. Science 353, 141–144 (2016). 27198674 10.1126/science.aaf2458

[29] J. Wang, Y. Zhang, P. Zhang, J. Hu, R.-B. Lin, Q. Deng, Z. Zeng, H. Xing, S. Deng, B. Chen, Optimizing pore space for flexible-robust metal-organic framework to boost trace acetylene removal. J. Am. Chem. Soc. 142, 9744–9751 (2020). 32406682 10.1021/jacs.0c02594

[30] R.-B. Lin, L. Li, H. Wu, H. Arman, B. Li, R.-G. Lin, W. Zhou, B. Chen, Optimized separation of acetylene from carbon dioxide and ethylene in a microporous material. J. Am. Chem. Soc. 139, 8022–8028 (2017). 28574717 10.1021/jacs.7b03850

[31] J. Shen, X. He, T. Ke, R. Krishna, J. M. van Baten, R. Chen, Z. Bao, H. Xing, M. Dincǎ, Z. Zhang, Simultaneous interlayer and intralayer space control in two-dimensional metal-organic frameworks for acetylene/ethylene separation. Nat. Commun. 11, 6259 (2020). 33288766 10.1038/s41467-020-20101-7 PMC7721749

[32] E. D. Bloch, W. L. Queen, R. Krishna, J. M. Zadrozny, C. M. Brown, J. R. Long, Hydrocarbon separations in a metal-organic framework with open iron (II) coordination sites. Science 335, 1606–1610 (2012). 22461607 10.1126/science.1217544

[33] L. Li, R.-B. Lin, R. Krishna, H. Li, S. Xiang, H. Wu, J. Li, W. Zhou, B. Chen, Ethane/ethylene separation in a metal-organic framework with iron-peroxo sites. Science 362, 443–446 (2018). 30361370 10.1126/science.aat0586

[34] O. T. Qazvini, R. Babarao, Z.-L. Shi, Y.-B. Zhang, S. G. Telfer, A robust ethane-trapping metal-organic framework with a high capacity for ethylene purification. J. Am. Chem. Soc. 141, 5014–5020 (2019). 30860821 10.1021/jacs.9b00913

[35] R.-B. Lin, H. Wu, L. Li, X.-L. Tang, Z. Li, J. Gao, H. Cui, W. Zhou, B. Chen, Boosting ethane/ethylene separation within isoreticular ultramicroporous metal–organic frameworks. J. Am. Chem. Soc. 140, 12940–12946 (2018). 30216725 10.1021/jacs.8b07563

[36] S. Chen, Y. Li, Z. Bu, F. Yang, J. Luo, Q. An, Z. Zeng, J. Wang, S. Deng, Boosting CO_2_-to-CO conversion on a robust single-atom copper decorated carbon catalyst by enhancing intermediate binding strength. J. Mater. Chem. A 9, 1705–1712 (2021).

[37] J. D. Yi, D. H. Si, R. Xie, Q. Yin, M. D. Zhang, Q. Wu, G. L. Chai, Y. B. Huang, R. Cao, Conductive two-dimensional phthalocyanine-based metal-organic framework nanosheets for efficient electroreduction of CO_2_. Angew. Chem. Int. Ed. 133, 17245–17251 (2021). 10.1002/anie.202104564 34033203

[38] Q.-L. Qian, X.-W. Gu, J. Pei, H.-M. Wen, H. Wu, W. Zhou, B. Li, G. Qian, A novel anion-pillared metal–organic framework for highly efficient separation of acetylene from ethylene and carbon dioxide. J. Mater. Chem. A 9, 9248–9255 (2021).

[39] M. Thommes, K. Kaneko, A. V. Neimark, J. P. Olivier, F. Rodriguez-Reinoso, J. Rouquerol, K. S. Sing, Physisorption of gases, with special reference to the evaluation of surface area and pore size distribution (IUPAC Technical Report). Pure Appl. Chem. 87, 1051–1069 (2015).

[40] P. Zhang, Y. Zhong, J. Ding, J. Wang, M. Xu, Q. Deng, Z. Zeng, S. Deng, A new choice of polymer precursor for solvent-free method: Preparation of N-enriched porous carbons for highly selective CO_2_ capture. Chem. Eng. J. 355, 963–973 (2019).

[41] P. Zhang, J. Wang, W. Fan, Y. Zhong, Y. Zhang, Q. Deng, Z. Zeng, S. Deng, Ultramicroporous carbons with extremely narrow pore size distribution via in-situ ionic activation for efficient gas-mixture separation. Chem. Eng. J. 375, 121931 (2019).

[42] P.-Q. Liao, W.-X. Zhang, J.-P. Zhang, X.-M. Chen, Efficient purification of ethene by an ethane-trapping metal-organic framework. Nat. Commun. 6, 8697 (2015). 26510376 10.1038/ncomms9697 PMC4846320

[43] Y. X. Wang, S. Yuan, Z. G. Hu, T. Kundu, J. Zhang, S. B. Peh, Y. D. Cheng, J. Q. Dong, D. Q. Yuan, H. C. Zhou, D. Zhao, Pore size reduction in zirconium metal-organic frameworks for ethylene/ethane separation. ACS Sustain. Chem. Eng. 7, 7118–7126 (2019).

[44] C. Gucuyener, J. van den Bergh, J. Gascon, F. Kapteijn, Ethane/ethene separation turned on its head: Selective ethane adsorption on the metal-organic framework ZIF-7 through a gate-opening mechanism. J. Am. Chem. Soc. 132, 17704–17706 (2010). 21114318 10.1021/ja1089765

[45] W. Yuan, X. Zhang, L. Li, Synthesis of zeolitic imidazolate framework-69 for adsorption separation of ethane and ethylene. J. Solid State Chem. 251, 198–203 (2017).

[46] L. Huang, D. P. Cao, Selective adsorption of olefin-paraffin on diamond-like frameworks: Diamondyne and PAF-302. J. Mater. Chem. A 1, 9433–9439 (2013).

[47] U. Böhme, B. Barth, C. Paula, A. Kuhnt, W. Schwieger, A. Mundstock, J. Caro, M. Hartmann, Ethene/ethane and propene/propane separation via the olefin and paraffin selective metal–organic framework adsorbents CPO-27 and ZIF-8. Langmuir 29, 8592–8600 (2013). 23802617 10.1021/la401471g

[48] D. Lv, R. Shi, Y. Chen, Y. Wu, H. Wu, H. Xi, Q. Xia, Z. Li, Selective adsorption of ethane over ethylene in PCN-245: Impacts of interpenetrated adsorbent. ACS Appl. Mater. Interfaces 10, 8366–8373 (2018). 29431991 10.1021/acsami.7b19414

[49] Y. Chen, H. Wu, D. Lv, R. Shi, Y. Chen, Q. Xia, Z. Li, Highly adsorptive separation of ethane/ethylene by an ethane-selective MOF MIL-142A. Ind. Eng. Chem. Res. 57, 4063–4069 (2018).

[50] H. Wu, Y. Chen, D. Lv, R. Shi, Y. Chen, Z. Li, Q. Xia, An indium-based ethane-trapping MOF for efficient selective separation of C_2_H_6_/C_2_H_4_ mixture. Sep. Purif. Technol. 212, 51–56 (2019).

[51] J. Pires, M. L. Pinto, V. K. Saini, Ethane selective IRMOF-8 and its significance in ethane-ethylene separation by adsorption. ACS Appl. Mater. Interfaces 6, 12093–12099 (2014). 25010787 10.1021/am502686g

[52] H. Xiang, Y. Shao, A. Ameen, H. Chen, W. Yang, P. Gorgojo, F. Siperstein, X. Fan, Q. Pan, Adsorptive separation of C_2_H_6_/C_2_H_4_ on metal-organic frameworks (MOFs) with pillared-layer structures. Sep. Purif. Technol. 242, 116819 (2020).

[53] W. Liang, F. Xu, X. Zhou, J. Xiao, Q. Xia, Y. Li, Z. Li, Ethane selective adsorbent Ni(bdc)(ted)_0.5_ with high uptake and its significance in adsorption separation of ethane and ethylene. Chem. Eng. Sci. 148, 275–281 (2016).

[54] Y. Chen, Z. Qiao, H. Wu, D. Lv, R. Shi, Q. Xia, J. Zhou, Z. Li, An ethane-trapping MOF PCN-250 for highly selective adsorption of ethane over ethylene. Chem. Eng. Sci. 175, 110–117 (2018).

[55] K.-J. Chen, D. G. Madden, S. Mukherjee, T. Pham, K. A. Forrest, A. Kumar, B. Space, J. Kong, Q.-Y. Zhang, M. J. Zaworotko, Synergistic sorbent separation for one-step ethylene purification from a four-component mixture. Science 366, 241–246 (2019). 31601769 10.1126/science.aax8666

[56] S. Mukherjee, N. Kumar, A. A. Bezrukov, K. Tan, T. Pham, K. A. Forrest, K. A. Oyekan, O. T. Qazvini, D. G. Madden, B. Space, M. J. Zaworotko, Amino-functionalised hybrid ultramicroporous materials that enable single-step ethylene purification from a ternary mixture. Angew. Chem. Int. Ed. 60, 10902–10909 (2021). 10.1002/anie.202100240 PMC8252428 33491848

[57] B. C. Smith, in *Fundamentals of Fourier Transform Infrared Spectroscopy* (CRC Press, ed. 2, 2011), p. 207.

[58] A. Dutta, Fourier transform infrared spectroscopy, in *Spectroscopic Methods for Nanomaterials Characterization* (Maharashtra Institute of Technology, 2017) pp. 73–93.

