# Supplementary material for: Synergistic binding sites in a hybrid ultramicroporous material for one-step ethylene purification from ternary C2 hydrocarbon mixtures
Source: Sci Adv. 2022 Jun 8;8(23):eabn9231. doi: 10.1126/sciadv.abn9231 (PMC9176739; doi:10.1126/sciadv.abn9231)
Supplement: Supplementary file 1 — Figs. S1 to S32 Tables S1 to S7 References [file sciadv.abn9231_sm.pdf]

Science Advances
AAAS

Supplementary Materials for

Synergistic binding sites in a hybrid ultramicroporous material for one-step ethylene purification from ternary C_2 hydrocarbon mixtures

Peixin Zhang *et al.*

Corresponding author: Jun Wang, jwang7@ncu.edu.cn

Sci. Adv. **8**, eabn9231 (2022)
DOI: 10.1126/sciadv.abn9231

This PDF file includes:

Figs. S1 to S32
Tables S1 to S7
References

Table S1. EXAFS fitting parameters at the Cu K-edge for $CuTiF_6$-TPPY samples （S_0^2=0.75, 0.86）

Sample	Shell	N^a	R(Å)^b	$\sigma^2\times10^3$(Å^2)^c	ΔE_0 (eV)^d	R factor
Cu foil	Cu-Cu	12*	2.54±0.01	8.9±0.3	4.5±0.5	0.002
CuPc	Cu-N	4.2±1.1	1.95±0.02	4.4±2.4	8.3±2.9	0.009
	Cu-C	4.3±1.4	2.94±0.03	7.2±5.2	7.0±4.4	
CuF_2	Cu-F	3.8±1.4	1.91±0.01	2.8±0.8	2.6±1.6	0.012
Cu1	Cu-N	5.8±0.8	1.97±0.01	4.5±1.3	-4.0±.6	0.003
Cu2	Cu-N	4*	1.92±0.04	18.1±5.0	-5.6±10.5	0.017
	Cu-F	2*	1.92±0.04	2.2±1.0	-6.5±12.5	

$^a N$: coordination numbers; $^b R$: bond distance; $^c\sigma^2$: Debye-Waller factors; ^{d} ΔE_0: the inner potential correction. R factor: goodness of fit.

In EXAFS fitting, the adjacent coordination elements in the periodic table are difficult to be precisely distinguished, as well as the Cu-N and Cu-F paths. For this reason, Cu is firstly only considered as one M-N shell, and the obtained results showed that the coordination number is close to 6. On this basis, Cu-F was introduced to limit the coordination number, the result of Cu2 and expected Cu-N_4F_2 was fitted. As shown in Table S1, the fitting parameters for the Cu-N_4F_2 coordination environment are reasonable.

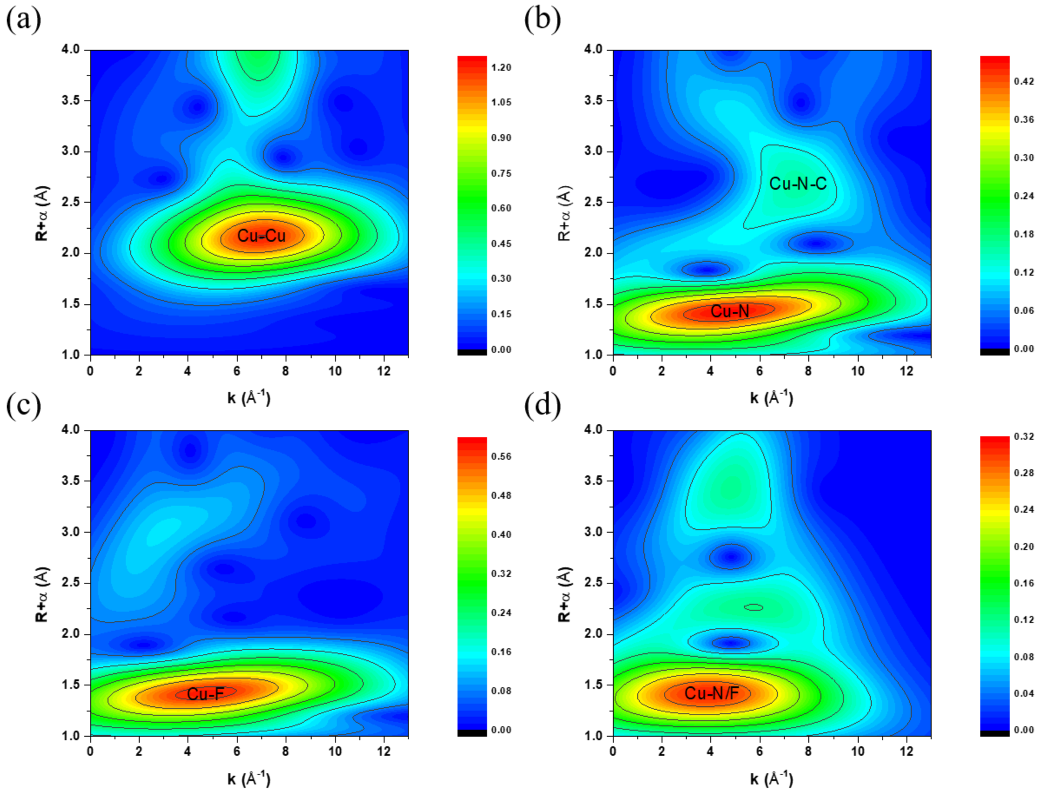

Fig. S1. The wavelet transform (WT) plots. The WT plots for (a) Cu foil, (b) CuPc, (c) CuF_2, and (d) $CuTiF_6$-TPPY.

Structure simulation

The structure model of $CuTiF_6$-TPPY was generated, based on the coordination mode by XAS data, using the Materials Studio suite of programs. The unit cell structures (e.g., cell parameters and atomic positions) of $CuTiF_6$-TPPY were calculated using the Forcite and Castep module. The Rietveld refinement, a software package for crystal determination from the XRD pattern, was performed to optimize the lattice parameters iteratively until the *w*Rp value converges. The pseudo-Voigt profile function was used for whole profile fitting and Berrar–Baldinozzi function was used for asymmetry correction during the refinement processes. Line broadening from crystallite size and lattice strain were both considered.

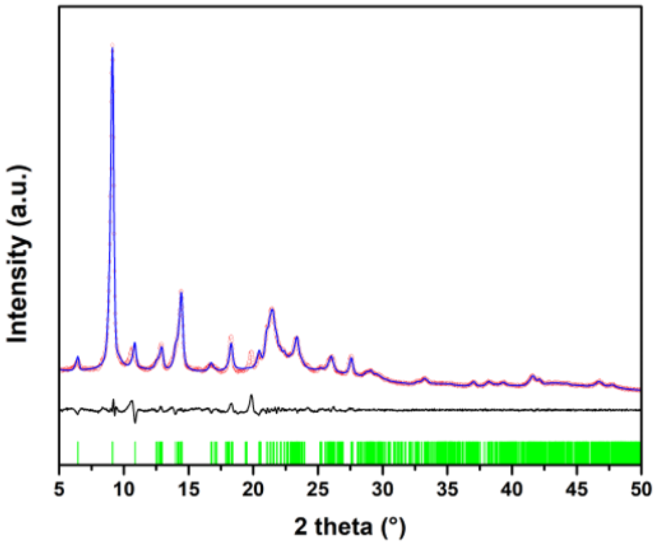

Fig. S2. PXRD Rietveld refinement. Rietveld refinement plot for $CuTiF_6$-TPPY.

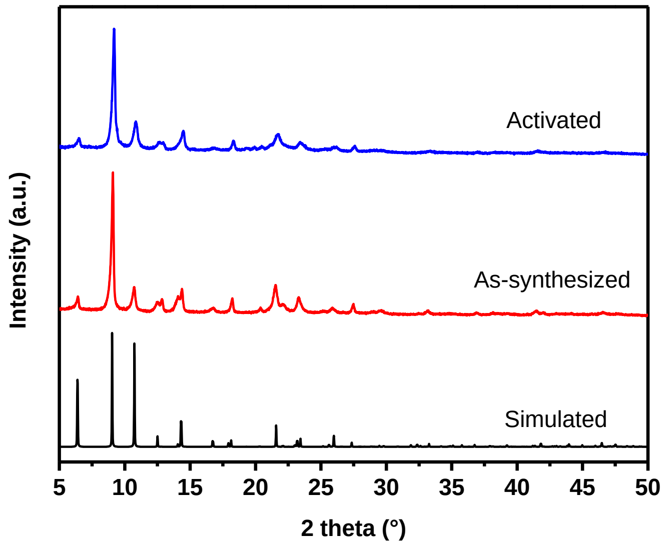

Fig. S3. PXRD patterns. PXRD patterns of simulated, as-synthesized, and activated $CuTiF_6$-TPPY.

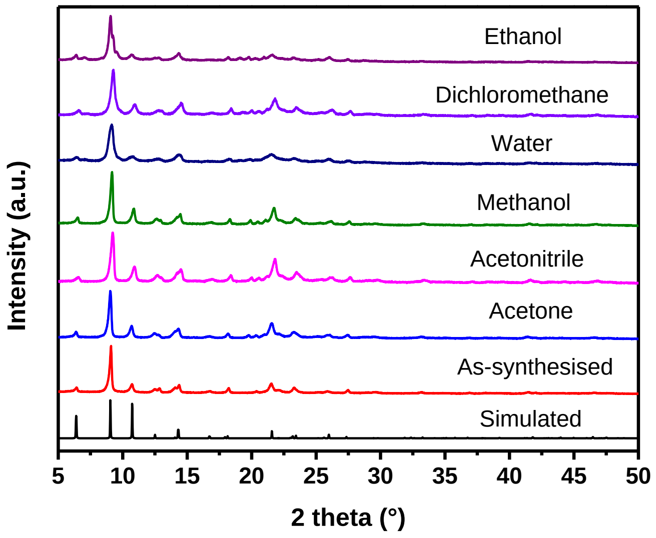

Fig. S4. PXRD patterns after treatments. PXRD patterns of $CuTiF_6$-TPPY after treatment in different solvents for one week.

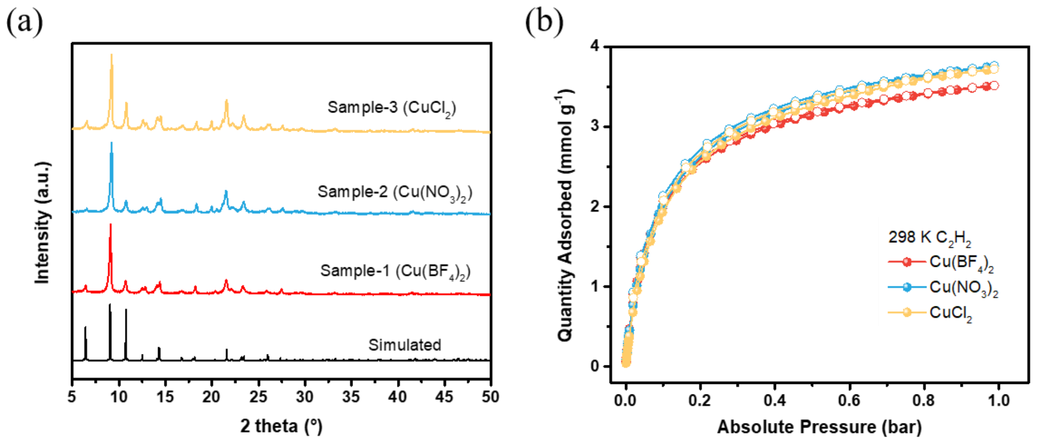

Fig. S5. Sample synthesis with different Cu salts and their C_2H_2 adsorption isotherms. (a) XRD patterns and (b) C_2H_2 isotherms at 298 K of $CuTiF_6$-TPPY using different Cu salts.

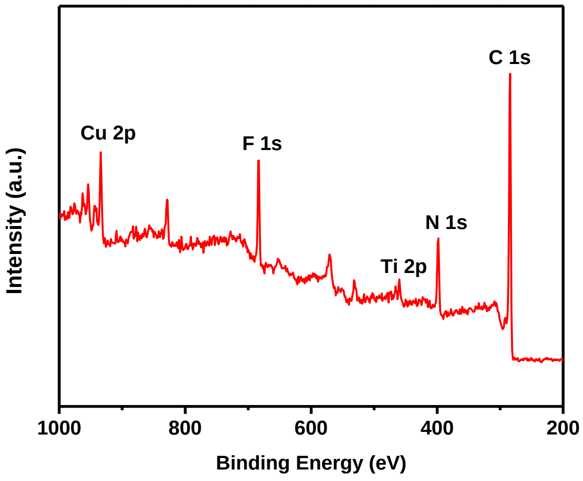

Fig. S6. XPS wide survey. XPS wide spectra of $CuTiF_6$-TPPY.

Table S2. Lattice parameters of the modeled structure of $CuTiF_6$-TPPY.

Unit cell parameters	$CuTiF_6$-TPPY
Formula	$C_{40}H_{26}N_8F_6TiCu$
Formula weight	843.42
Crystal system	Orthorhombic
Space group	P222
a (Å)	13.85785
b (Å)	13.78813
c (Å)	8.23169
α (°)	90
β (°)	90
γ (°)	90
V (Å^3)	1572.86
Z	1
D_{calcd} (g cm^{-3})	0.8912
Rp [a]	0.0213
R_{wp} [b]	0.0326
GOF	1.06

[a] $R_p = \Sigma|cY_{sim}(2\theta_i) - I_{exp}(2\theta_i) + Y_{back}(2\theta_i)|/\Sigma|I_{exp}(2\theta_i)|$.

[b] $R_{wp} = \{w_p[cY_{sim}(2\theta_i) - I_{exp}(2\theta_i) + Y_{back}(2\theta_i)]^2/\Sigma w_p[I_{exp}(2\theta_i)]^2\}^{1/2}$, and $w_p = 1/I_{exp}(2\theta_i)$.

Table S3. The elemental analysis for $CuTiF_6$-TPPY

$CuTiF_6$-TPPY	Cu	Ti	Si	F	C	N	H
Theoretical value	7.64	5.73	/	13.5	56.9	13.3	3.08
ICP-OES (wt%)	9.07	7.06	/	/	/	/	/
Element analysis (wt%)	/	/	/	/	53.9	11.3	3.95
XPS	3.02	2.00	/	8.61	73.59	12.78	/

Table S4. Fractional atomic coordinates for the unit cell of $CuTiF_6$-TPPY (*P222* model obtained from the Rietveld refinement, R_p = 0.0213 R_{wp} =0.0326).

	a= 13.85785<br>alpha = 90.000	b = 13.78813<br>beta = 90.000	c = 8.23169<br>gamma = 90.000
C	0.4511	0.8092	0.4986
C	0.4213	0.7081	0.5001
C	0.3239	0.6783	0.4991
C	0.2479	0.7541	0.4992
C	0.2417	0.8223	0.6270
C	0.1695	0.8921	0.6241
N	0.1040	0.8960	0.5005
C	0.1087	0.8309	0.3763
C	0.1794	0.7596	0.3721
H	0.4034	0.8716	0.4954
H	0.2924	0.8206	0.7299
H	0.1613	0.9450	0.7231
H	0.0556	0.8386	0.2777
H	0.1815	0.7092	0.2687
F	-0.0963	0.9033	0.9998
C	0.1934	0.5496	0.5006
C	0.2914	0.5819	0.4989
H	0.1313	0.5971	0.5027
N	0.5000	0.6476	0.5000
N	0.3482	0.5000	0.5000
H	0.4223	0.5000	0.5000
Cu	0.0000	1.0000	0.5000
Ti	0.0000	1.0000	1.0000
F	0.0000	1.0000	0.7662

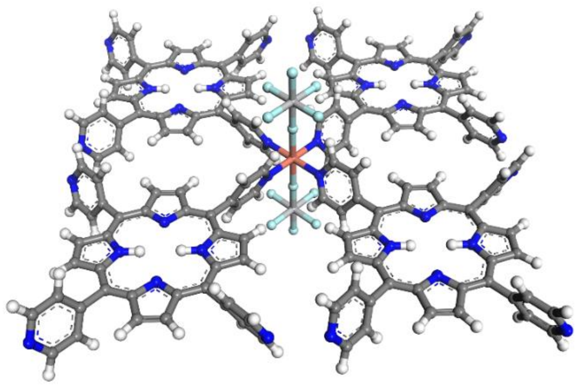

Fig. S7. Crystal coordination mode. The coordination mode of Cu, TiF_6^{2-}, and TPPY.

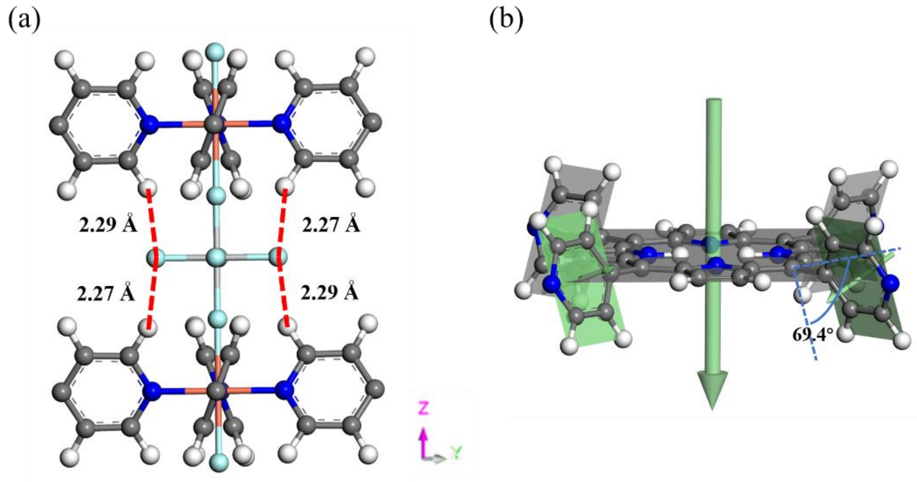

Fig. S8. The interactions and rotations of TPPY. (a) The hydrogen bond formed between TiF_6^{2-} pillars and pyridine rings, (b) the rotation angle of pyridine rings in $CuTiF_6$-TPPY.

(a) (b)

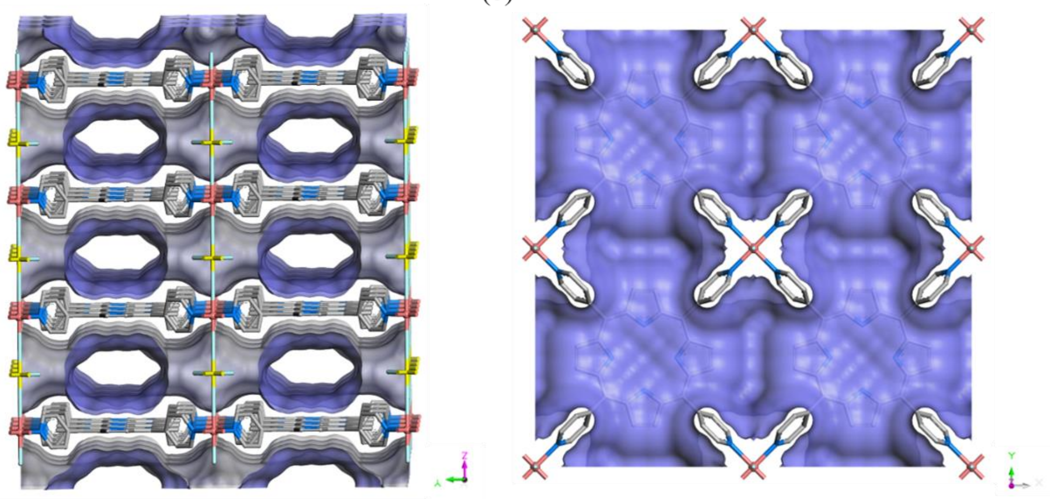

Fig. S9. Pore structures of $CuTiF_6$-TPPY. (a) 3D structures showing uniform one-dimensional channels and (b) the pore aperture sectional drawing in the *x, y* plane of $CuTiF_6$-TPPY.

(a) (b)

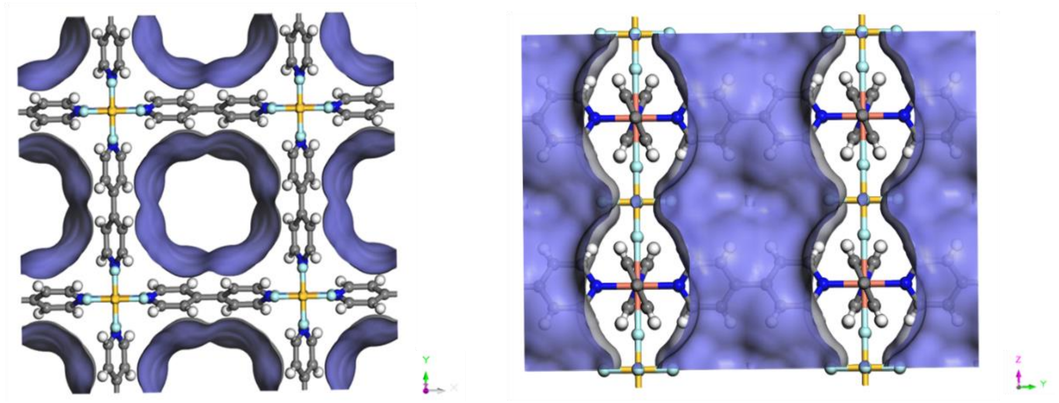

Fig. S10. Pore structures of SIFSIX-1-Cu. (a) 3D structures showing uniform one-dimensional channels and (b) the pore aperture sectional drawing in the *x, y* plane of SIFSIX-1-Cu.

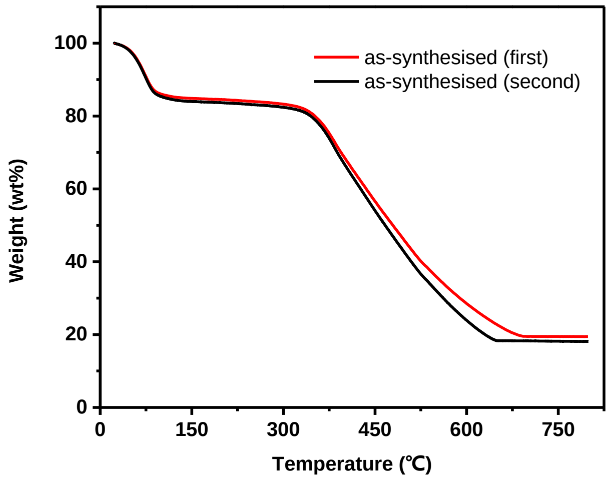

Fig. S11. TGA curves. TGA curve of the as-synthesized $CuTiF_6$-TPPY.

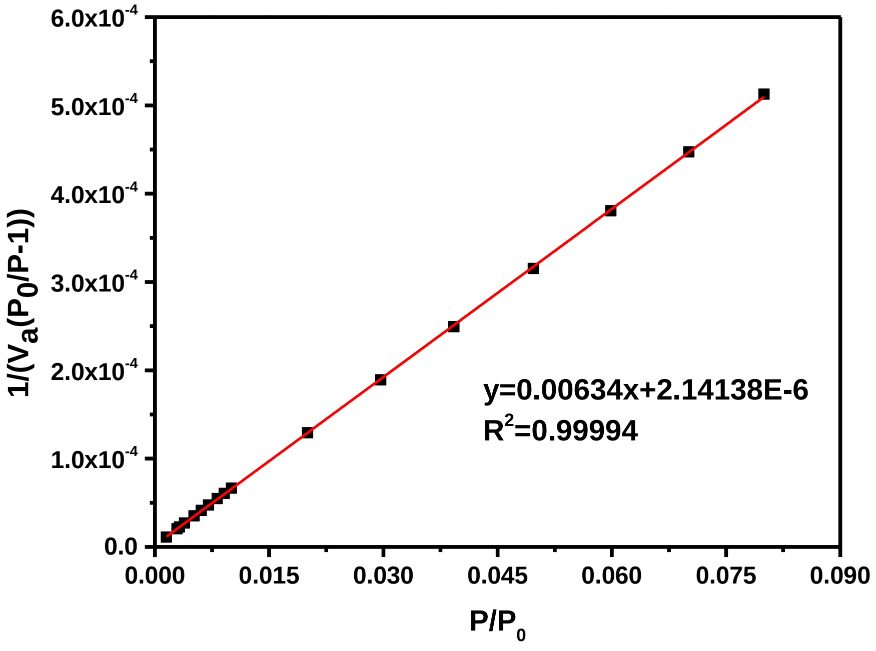

Fig. S12. BET calculation plot. BET calculation plot for $CuTIF_6$-TPPY based on its corresponding N_2 adsorption isotherm at 77 K.

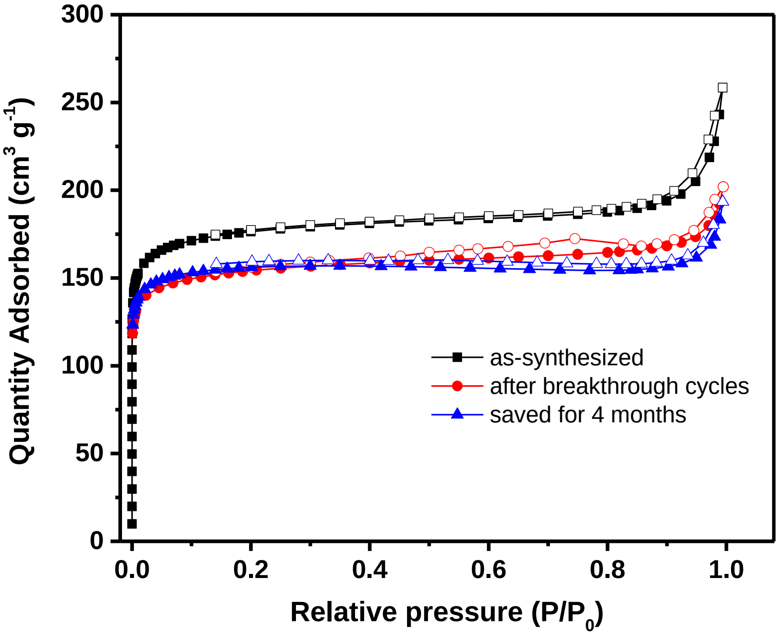

Fig. S13. N_2 adsorption isotherms at 77 K. N_2 adsorption isotherms of $CuTiF_6$-TPPY at 77 K after treating in different conditions.

Isotherm fitting

The pure-component isotherms of C_2H_2, C_2H_4 and C_2H_6 were fitted using single-site Langmuir-Freundlich model for full range of pressure (0~1 bar).

$$q = q_{sat} \frac{bp^v}{1 + bp^v}$$

Here, p is the pressure of the bulk gas at equilibrium with the adsorbed phase (bar), q is the adsorbed amount per mass of adsorbent (mmol g^{-1}), q_{sat} is the saturation capacities (mmol g^{-1}), b is the affinity coefficient (bar^{-1}), and v represent the deviation from an ideal homogeneous surface.

Table S5. Single-site Langmuir-Freundlich parameters of different gases on $CuTiF_6$-TPPY.

Gas	T<br>K	q_{sat}<br>mol kg^{-1}	b<br>bar^{-1}	v<br>dimensionless
C_2H_2	273	4.63989	8.23274	0.70065
	288	4.16236	9.63506	0.85908
	298	3.98252	7.03971	0.85311
C_2H_4	273	3.61553	5.28016	0.82235
	288	3.43124	4.44434	0.92472
	298	3.27381	2.73929	0.86244
C_2H_6	273	4.06598	8.00487	0.7914
	288	4.13356	4.05425	0.74243
	298	3.58502	3.49011	0.77377

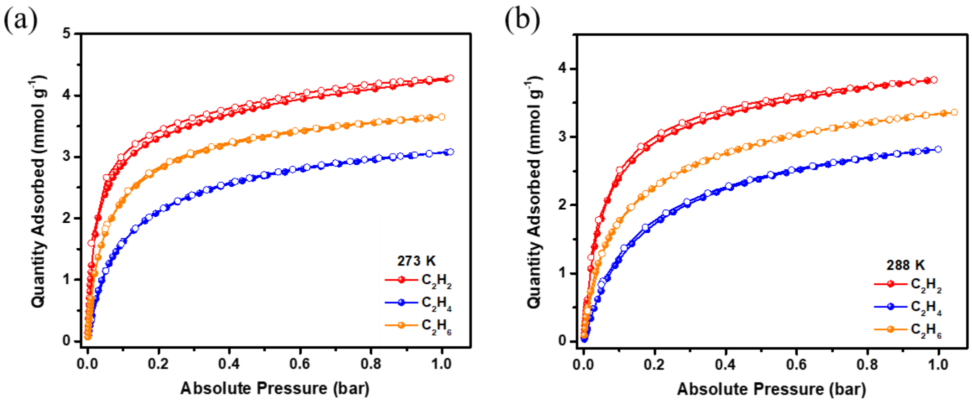

Fig. S14. C2 adsorption isotherms for $CuTiF_6$-TPPY at 273 and 288 K. The C_2H_2, C_2H_4, and C_2H_6 adsorption isotherms on $CuTiF_6$-TPPY at (a) 273 K and (b) 288 K.

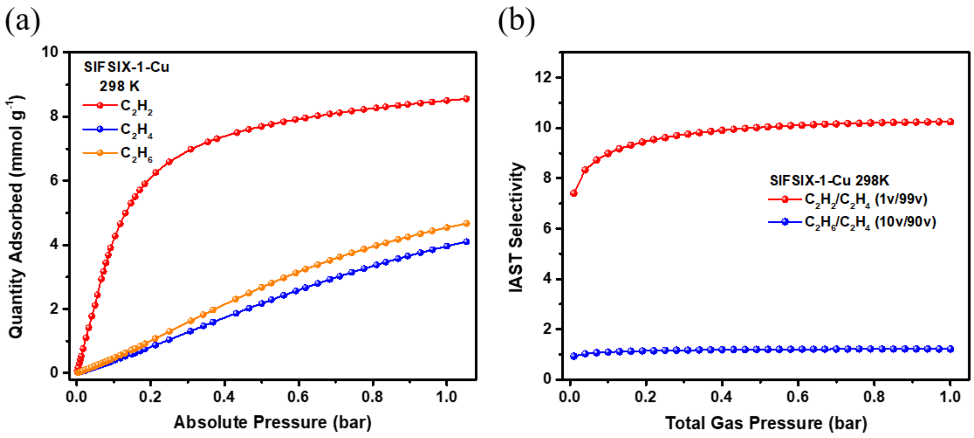

Fig. S15. C2 adsorption isotherms and IAST plot for SIFSIX-1-Cu. (a) C_2H_2, C_2H_4, and C_2H_6 adsorption isotherms on SIFSIX-1-Cu at 298 K, and (b) IAST selectivity of SIFSIX-1-Cu at different binary mixture compositions at 298 K.

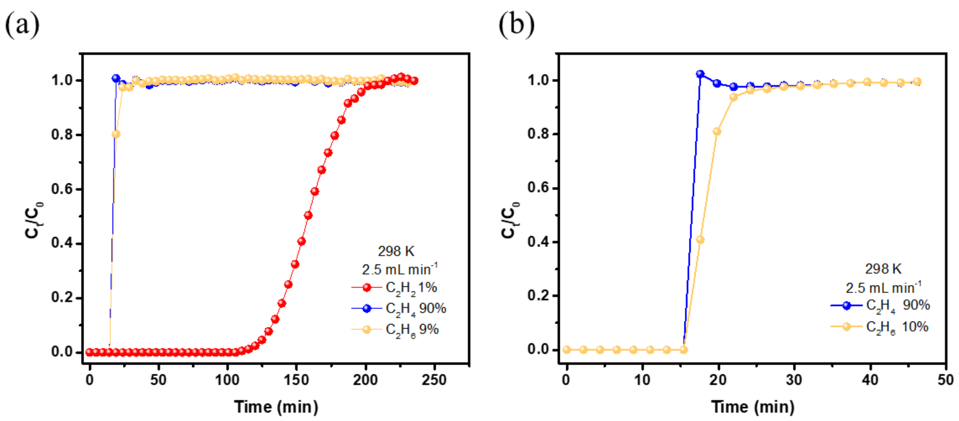

Fig. S16. Breakthrough curves for SIFSIX-1-Cu. The breakthrough curve of (a) $C_2H_2/C_2H_6/C_2H_4$ (1/9/90, *v/v/v*) and (b) C_2H_6/C_2H_4 (10/90, *v/v*) for SIFSIX-1-Cu (0.8 g) with a flow rate of 2.5 mL/min at 298 K.

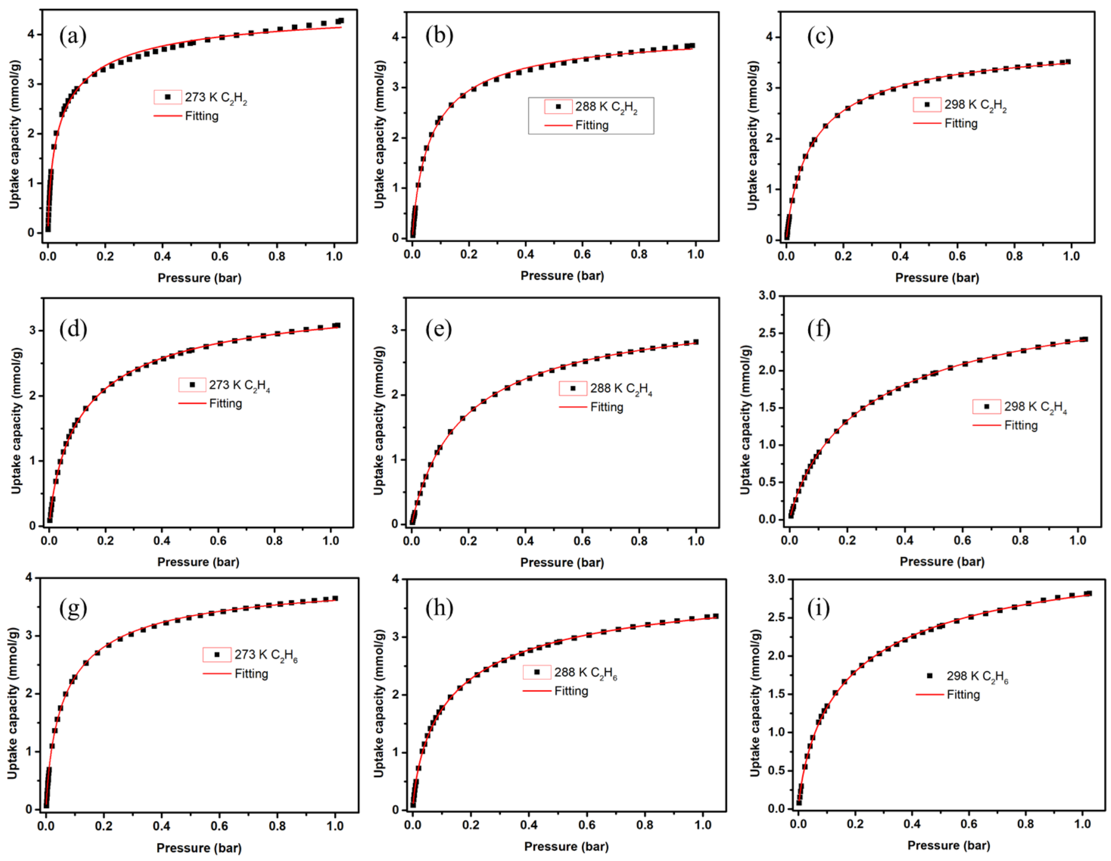

Fig. S17. The Langmuir-Freundlich fittings of C2 isotherms. The corresponding Langmuir-Freundlich fittings of C_2H_2, C_2H_4, and C_2H_6 adsorption isotherms on $CuTiF_6$-TPPY at different temperatures.

IAST calculations

The selectivity of the preferential adsorption of component 1 over component 2 in a mixture containing 1 and 2 can be formally defined as:

$$S = \frac{x_1/y_1}{x_2/y_2}$$

In the above equation, x_1 and y_1 (x_2 and y_2) are the molar fractions of component 1 (component 2) in the adsorbed and bulk phases, respectively. We calculated the values of x_1 and x_2 using the Ideal Adsorbed Solution Theory (IAST) of Myers and Prausnitz.

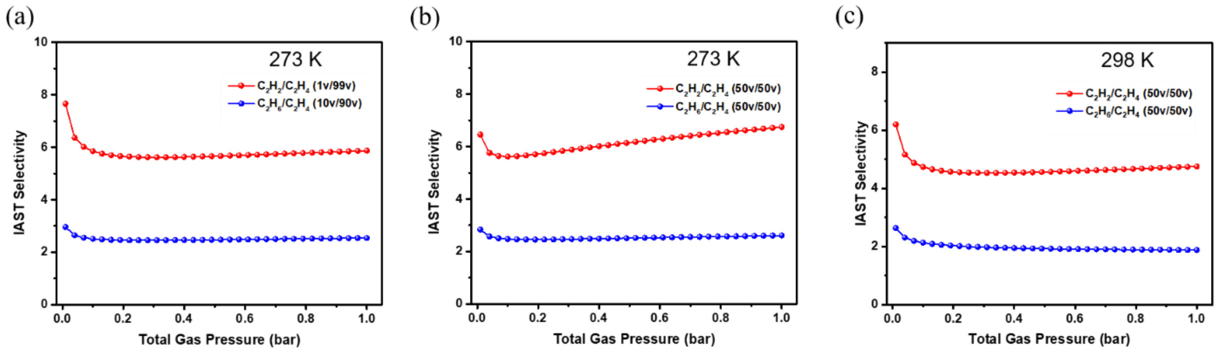

Fig. S18. IAST selectivity plots. IAST selectivity of $CuTiF_6$-TPPY at different binary mixture compositions and temperatures.

Isosteric heat of adsorption

The isosteric heat of C_2H_2, C_2H_4, and C_2H_6 adsorption, Q_{st}, defined as

$$Q_{st} = RT^2 \left(\frac{\partial In\ P}{\partial T} \right)_q$$

were determined using the pure component isotherm fits using the Clausius-Clapeyron equation. where Q_{st} (kJ/mol) is the isosteric heat of adsorption, T (K) is the temperature, P (bar) is the pressure, R is the gas constant, and q (mmol/g) is the adsorbed amount.

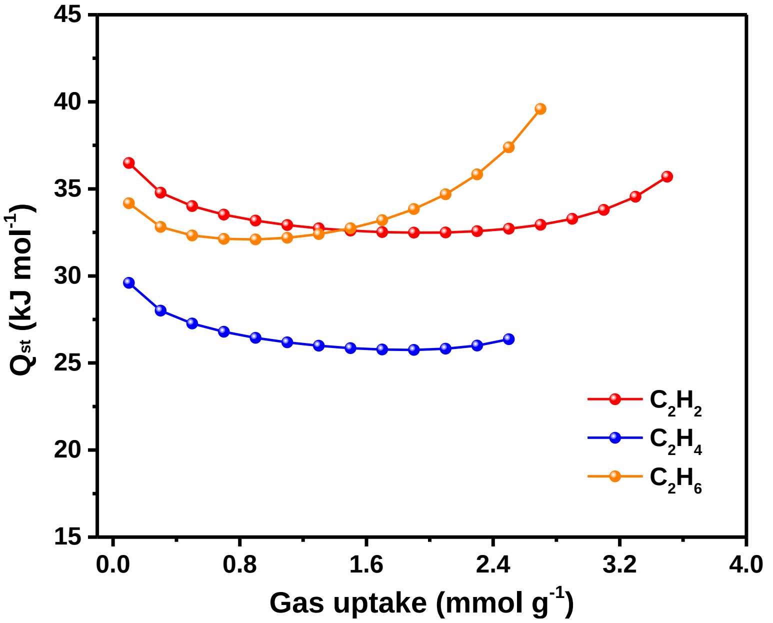

Fig. S19. C2 adsorption heats. C_2H_2, C_2H_4, and C_2H_6 isosteric adsorption heats on $CuTiF_6$-TPPY.

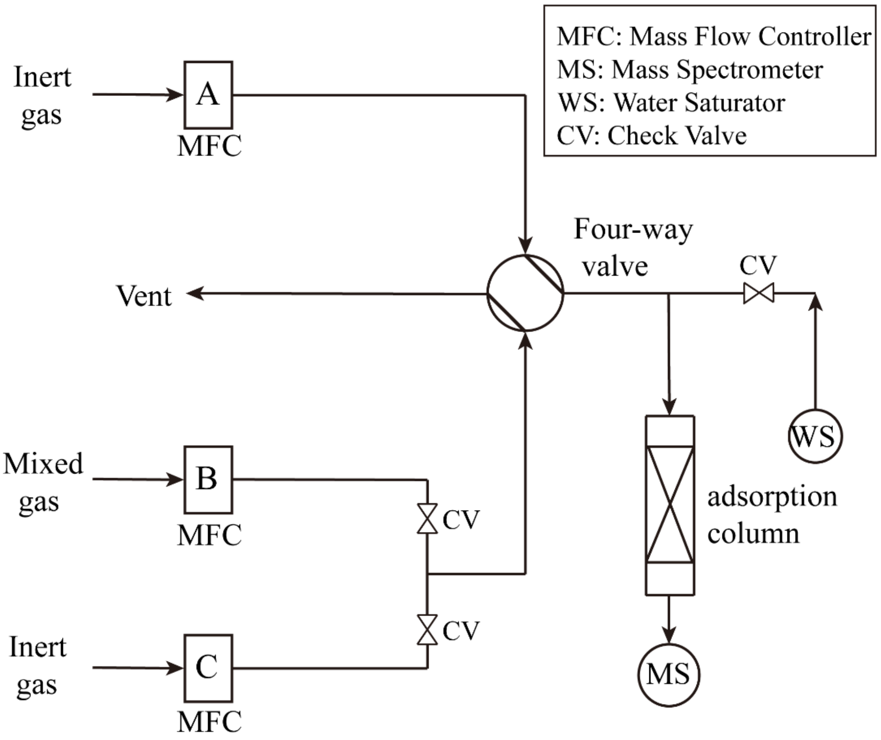

Fig. S20. Breakthrough apparatus. Representation of the column breakthrough experiment.

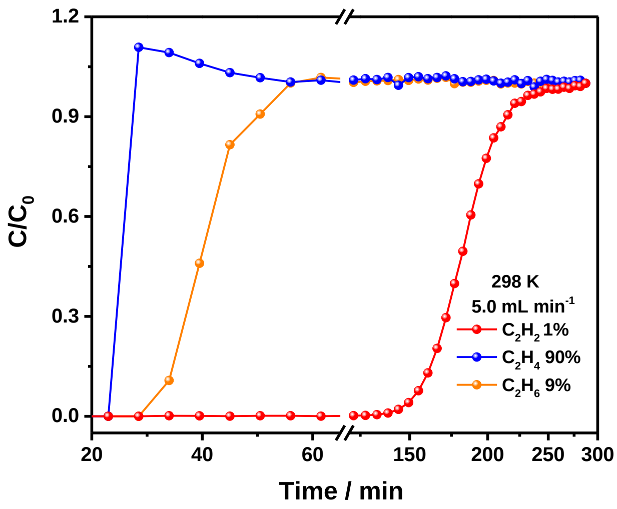

Fig. S21. Breakthrough curve for $CuTiF_6$-TPPY. The breakthrough curve of $C_2H_2/C_2H_6/C_2H_4$ (1/9/90, *v/v/v*) for $CuTiF_6$-TPPY with the flow rates of 5.0 mL/min at 298 K.

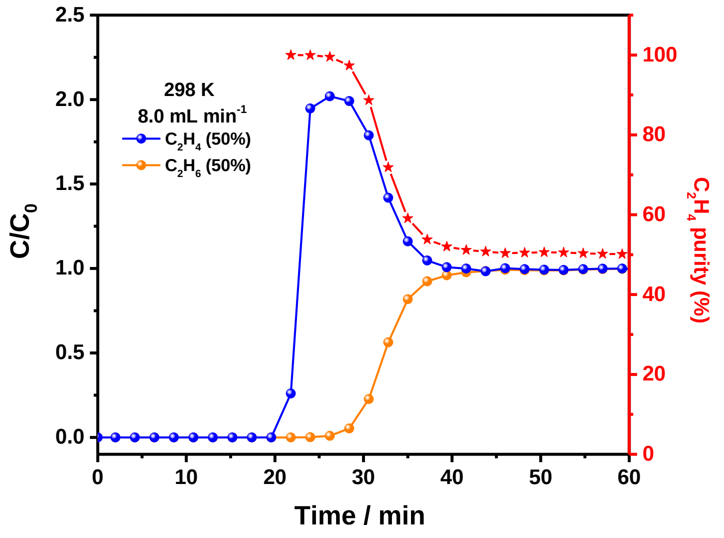

Fig. S22. Breakthrough curve for CuTiF$_6$-TPPY. The breakthrough curve of C_2H_6/C_2H_4 (50/50, *v/v*) for CuTiF$_6$-TPPY at the flow rate of 8.0 mL/min at 298 K.

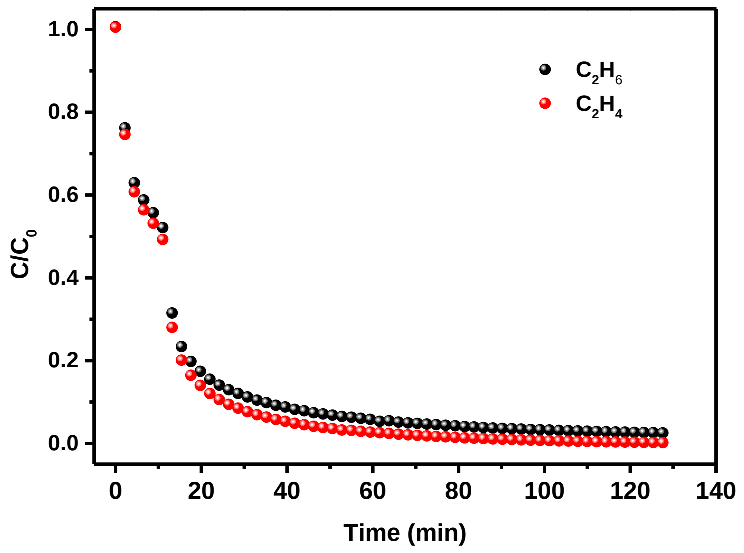

Fig. S23. Desorption curves for CuTiF$_6$-TPPY. The signals of desorbed C_2H_6 and C_2H_4 for CuTiF$_6$-TPPY (1.8 g) during the regeneration process under a He flow rate of 20 mL/min at 298 K.

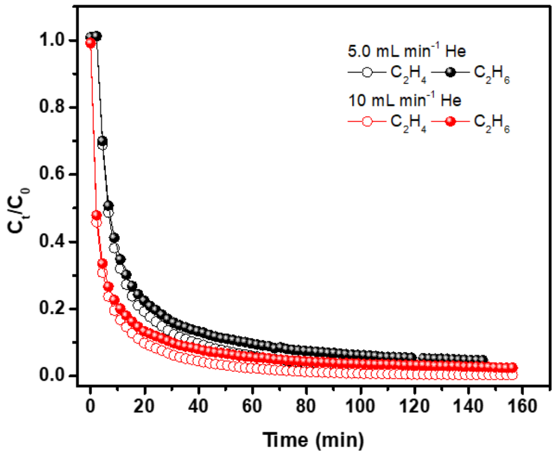

Fig. S24. Desorption curves for CuTiF$_6$-TPPY. The signals of desorbed C_2H_6 and C_2H_4 during the regeneration process from the adsorption column of CuTiF$_6$-TPPY (1.3 g).

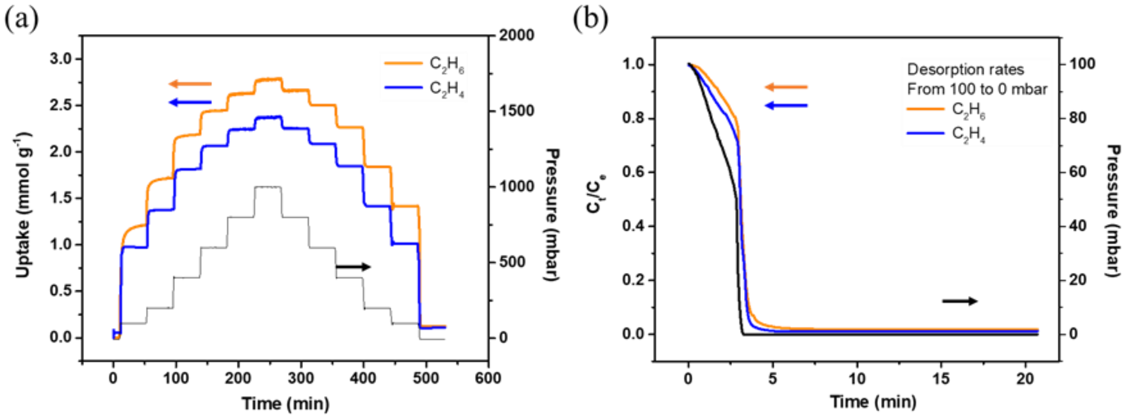

Fig. S25. Adsorption and desorption kinetics of C_2H_4 and C_2H_6. (a) Time-dependent gas uptake profiles of CuTiF$_6$-TPPY at pressures up to 1000 mbar with a raising rate of 100 mbar min^{-1} and kept for 60 min to reach full adsorption equilibriums and (b) desorption rate profiles for C_2H_4 and C_2H_6 on CuTiF$_6$-TPPY (from 100 to 0 mbar) at 298 K.

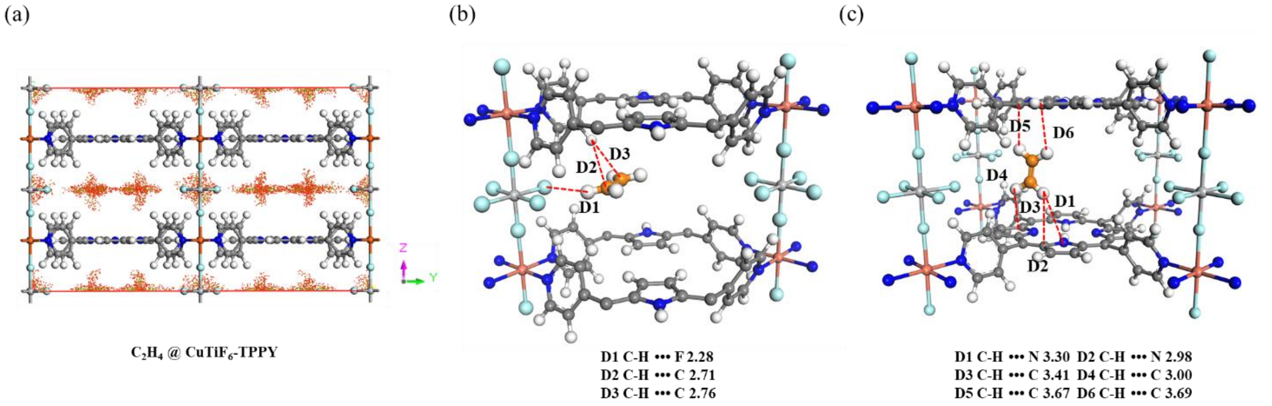

Fig. S26. Distribution density and binding sites for C_2H_4. (a) Computational simulations for the density distribution of C_2H_4 on $CuTiF_6$-TPPY at 100 kPa and 298 K, (b) (c) C_2H_4 binding sites in $CuTiF_6$-TPPY. The closest contacts between framework atoms and the gas molecules are defined by the distances (in Å) and the distances include the van der Waals radius. (Framework: C, grey-80%; H, white; N, blue; F, cyan; Cu, pink; Ti, silvery, Gas: C, orange; H, white).

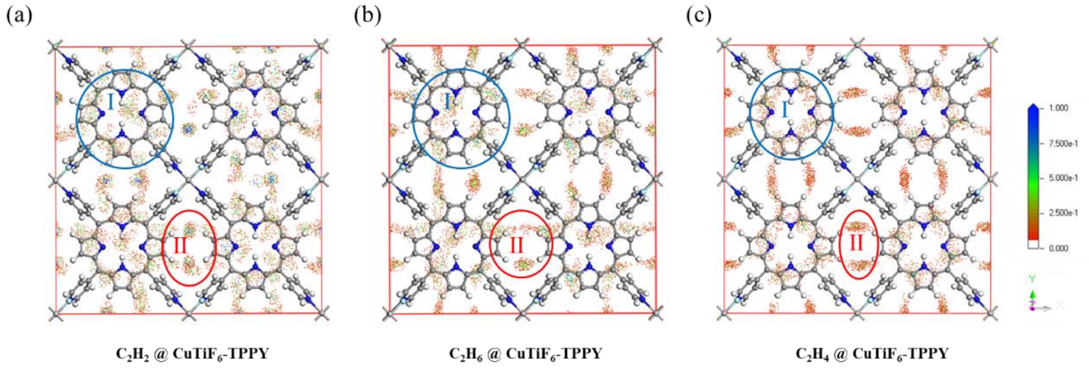

Fig. S27. C2 distribution densities. Computational simulations for the distribution density of (a) C_2H_2, (b) C_2H_6, and (c) C_2H_4 on $CuTiF_6$-TPPY at 100 kPa and 298 K.

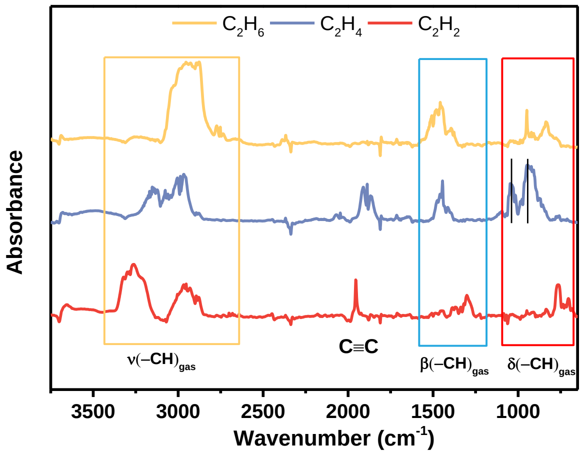

Fig. S28. *In-situ* IR spectrum. *In-situ* IR spectrum of activated CuTiF$_6$-TPPY sample exposed to C_2H_2, C_2H_4, or C_2H_6.

The three characteristic bands of ν(-CH), β-(CH), and δ(-CH) for C2 gases were detected, indicating the adsorption of all C2 gases in CuTiF$_6$-TPPY. The interactions between C2 gases and TiF_6^{2-} anion in CuTiF$_6$-TPPY cannot be directly characterized by IR spectroscopy because the vibrations of TiF_6^{2-} occur below 650 cm^{-1} that beyond the detection range of the infrared MCT-A detector. Nevertheless, some evidence for the interactions between gas molecules and TiF_6^{2-} anions were observed, because TiF_6^{2-} anions have strong electronegativity and can form strong hydrogen bonding with C2 hydrocarbons.

As for C_2H_2 adsorption, the stretching band ν(-CH) of adsorbed C_2H_2 appeared at a lower frequency of ~2950 cm^{-1} besides the frequency at ~3260 cm^{-1}, which was induced by the formation of hydrogen bonding between C_2H_2 and TiF_6^{2-} anions. The asymmetrical C≡C stretching band appears at a lower frequency of ~1950 cm^{-1} rather than 2100~2140 cm^{-1} also confirmed the formation of hydrogen bonding between C_2H_2 and TiF_6^{2-} anions (*56*). For C_2H_6 adsorption, the stretching band ν(-CH) of adsorbed C_2H_6 appeared at the lower frequency of 2800~3075 cm^{-1} implies the interactions between C_2H_6 and TiF_6^{2-} anions. The stretching bands of β-(CH) and δ(-CH) of adsorbed C_2H_6 were detected without frequency shifts, indicating that C_2H_6 also interacted with the aromatic skeleton of CuTiF$_6$-TPPY. As for C_2H_4, the δ(-CH) bending vibration bands appeared at ~1010 cm^{-1} and ~950 cm^{-1} belong to the absorption

peak of RCH=CH2 (*57,58*), no other interaction can be observed.

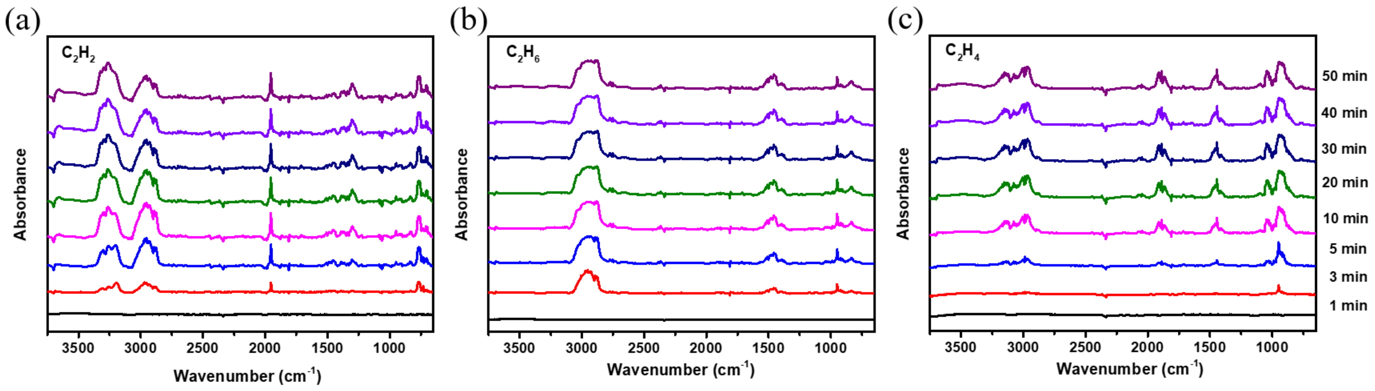

Fig. S29. Time-dependent *In-situ* IR spectra. *In-situ* IR spectra showing adsorbed (a) C_2H_2, (b) C_2H_6, and (c) C_2H_4 in CuTiF$_6$-TPPY with different exposure times.

To check the dependence of these spectral changes on the loading amount of guest molecules inside MOFs, time-dependent *in-situ* IR spectra with C2 gas-loadings were conducted. As shown in Fig. S29, the characteristic absorption peaks of C_2H_2 (ν(-CH) and asymmetrical C≡C stretching) and C_2H_6 (ν(-CH)) appeared at 3 min. In contrast, the stretching bands of adsorbed C_2H_4 were detected at 10 min. These results indicated that C_2H_2 and C_2H_6 can be adsorbed in a stronger and faster manner than C_2H_4 by CuTiF$_6$-TPPY.

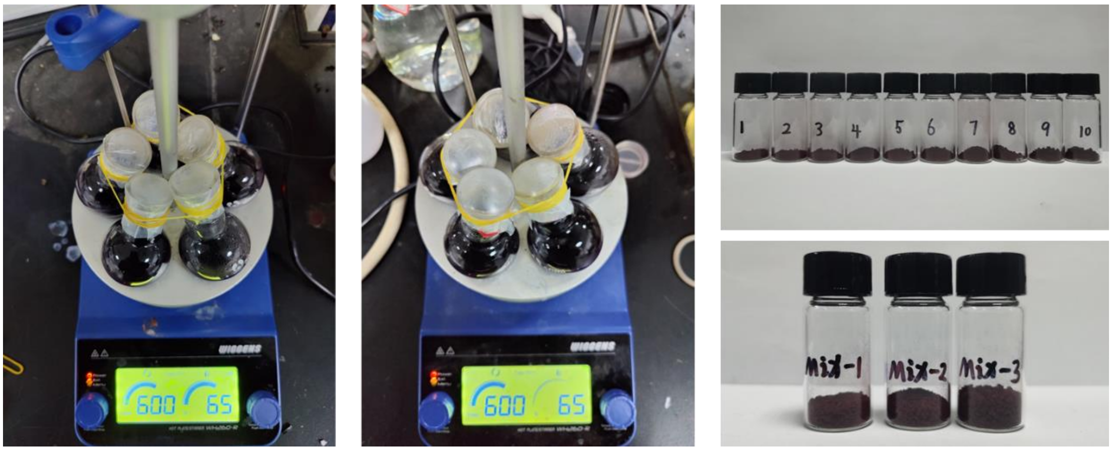

Fig. S30. Reproducibility of synthesis process. Images of different batches and blended mixtures of $CuTiF_6$-TPPY samples.

To verify the reproducibility of $CuTiF_6$-TPPY and its separation performances, we have parallelly synthesized ten batches of $CuTiF_6$-TPPY and measured their adsorptive separation performances. Due to the extremely time-consuming process for evaluating individual adsorption isotherms of 10-batch samples, we have blended samples 1-3 as labeled as “Mixture-1”, samples 4-6 as “Mixture-2”, and samples 7-10 as “Mixture-3” (Fig. S30).

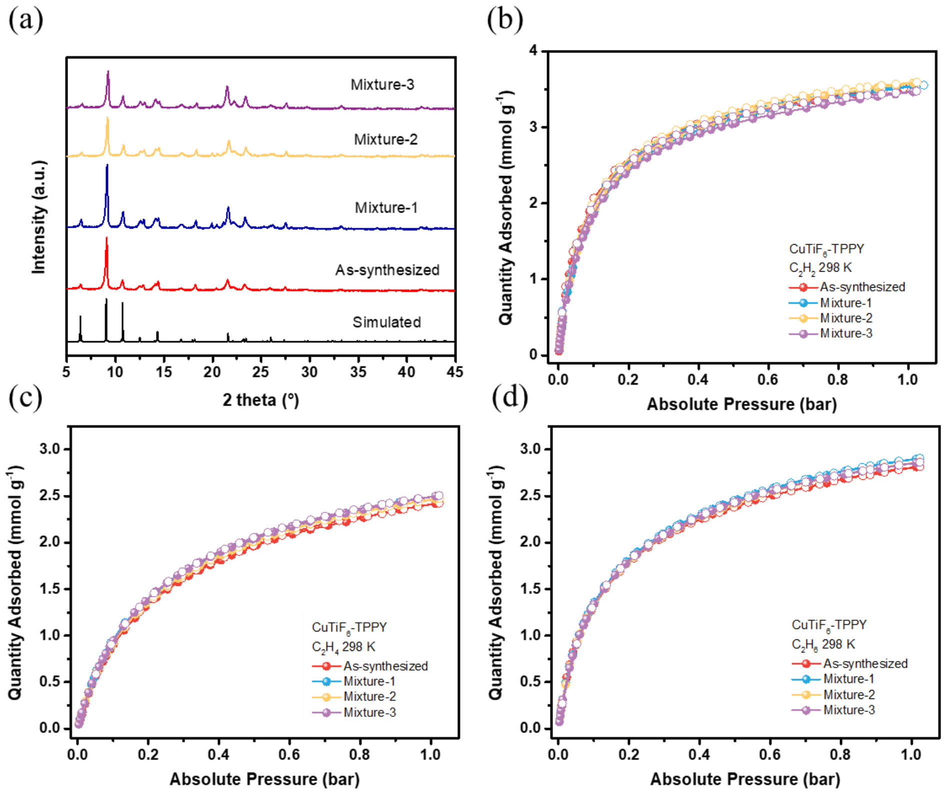

Fig. S31. Structure and adsorption properties of blended samples. (a) XRD patterns and adsorption isotherms of (b) C_2H_2, (c) C_2H_4, and (d) C_2H_6 on blended samples.

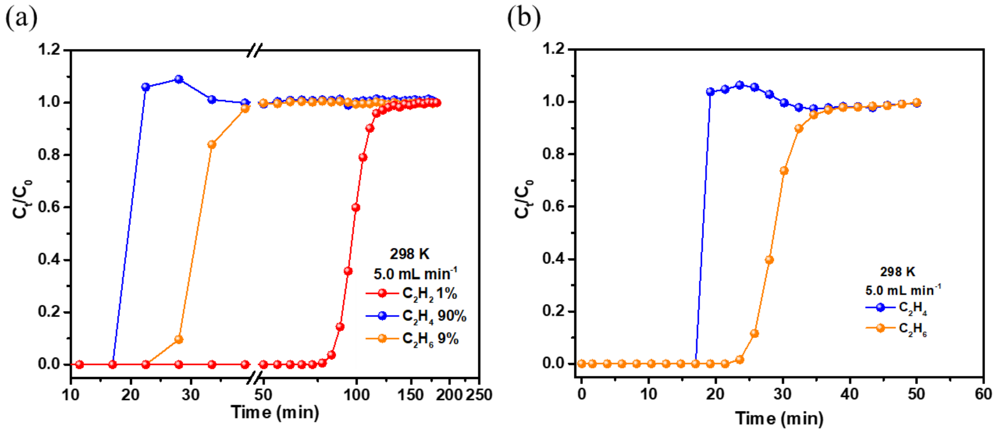

Fig. S32. Breakthrough curves for blended samples. The breakthrough curves of (a) $C_2H_2/C_2H_6/C_2H_4$ (1/9/90, *v*/*v*/*v*) and (b) C_2H_6/C_2H_4 (10/90, *v*/*v*) for $CuTiF_6$-TPPY (1.3 g) with a flow rate of 5.0 mL/min.

Table S6. Comparison of the equilibrium adsorption capacity and selectivity of the selected C_2H_6-selective MOFs at 298 K and 1 bar.

Adsorbents	Adsorption uptake (mmol/g)		Q_{st} (kJ/mol) at zero coverage		IAST Selectivity	Reference
	C_2H_6	C_2H_4	C_2H_6	C_2H_4		
UiO-66-ADC	1.7	1.8	36.0	36.0	1.8	(*43*)
MAF-49	1.7	1.7	56.7	45.5	2.7	(*42*)
ZIF-7	1.9	1.9	27.4	24.6	1.8	(*44*)
$Cu(Qc)_2$	1.9	0.8	28.1	25.2	3.5	(*35*)
ZIF-69	2.2	1.8	25.5	22.9	1.7	(*45*)
PAF-302	3.1	2.2	33.9	33.9	1.4	(46)
ZIF-8	3.2	2.0	21.5	16.0	2.0	(47)
PCN-245	3.3	2.4	23.0	20.5	1.8	(48)
$Fe_2(O_2)$(dobdc)	3.3	2.7	66.8	37.6	4.4	(*33*)
MIL-142A	3.8	2.9	27.3	26.1	1.5	(49)
In-soc-MOF-1	4.0	3.7	28.4	25.2	1.4	(*50*)
IRMOF-8	4.1	3.1	52.7	49.8	1.8	(*51*)
MUF-15	4.7	4.2	29.2	28.2	2.0	(*34*)
Ni2-a	4.8	4.6	33.5	31.7	1.4	(*52*)
Ni(bdc)$(ted)_{0.5}$	5.0	3.4	21.5	18.4	2.0	(53)
PCN-250	5.2	4.2	23.6	21.1	1.9	(*54*)
Ni1-a	6.6	6.0	33.6	32.2	1.5	(52)
Zn-atz-ipa	1.76	1.75	45.8	40	2.0	(55)
$CuTiF_6$-TPPY	2.82	2.42	34.2	29.6	2.12	This work

Table S7. Comparison of the equilibrium adsorption capacity of the selected C_2H_2 and C_2H_6 simultaneous adsorption MOFs at 298 K and 1 bar.

Adsorbents	Adsorption uptake (mmol/g)			Uptake ratio (298 K 1 bar)		IAST Selectivity		Reference
	C_2H_2	C_2H_6	C_2H_4	C_2H_2/ C_2H_4	C_2H_6/ C_2H_4	C_2H_2/C_2H_4 (50/50)	C_2H_6/C_2H_4 (50/50)	
ZJNU-7	5.04	4.13	3.80	1.33	1.09	1.68	1.56	(*25*)
ZJNU-115	4.73	4.20	3.75	1.26	1.12	2.05	1.56	(*24*)
NPU-1	5.10	4.50	4.20	1.21	1.07	1.4	1.32	(*20*)
NPU-2	3.99	4.42	3.42	1.17	1.29	1.25	1.52	(*20*)
NPU-3	2.19	3.33	2.19	1.0	1.52	1.32	3.21	(*20*)
TJT-100	4.46	3.70	3.44	1.3	1.08	1.8	1.2	(*5*)
Azole-Th-1	3.51	4.42	3.56	0.99	1.24	1.09	1.46	(*21*)
NUM-9a (313 K)	1.98	2.06	1.79	1.11	1.15	1.50	1.62	(*23*)
MOF-525	2.65	2.71	2.11	1.26	1.28	1.45	1.22	(22)
MOF-525(Co)	2.62	2.22	1.92	1.36	1.16	1.95	1.1	(*22*)
UPC-612	3.01	3.57	2.79	1.08	1.28	1.08	1.4	(*22*)
UPC-613	2.83	2.55	2.30	1.23	1.11	1.39	1.48	(*22*)
MIL-125	7.05	4.83	3.98	1.77	1.21	2.32	1.21	(*27*)
NH_2-MIL-125	7.82	4.69	4.41	1.78	1.06	3.75	1.18	(*27*)
ZSTU-2	3.11	2.73	2.35	1.32	1.16	2.36	1.62	(*27*)
$CuTiF_6$-TPPY	3.62	2.82	2.42	1.50	1.17	5.47	2.12	This work

REFERENCES AND NOTES

1. D. S. Sholl, R. P. Lively, Seven chemical separations to change the world. *Nature* **532**, 435–437 (2016).

2. J. Y. Lin, Molecular sieves for gas separation. *Science* **353**, 121–122 (2016).

3. I. Amghizar, L. A. Vandewalle, K. M. Van Geem, G. B. Marin, New trends in olefin production. *Engineering* **3**, 171–178 (2017).

4. T. Ren, M. Patel, K. Blok, Olefins from conventional and heavy feedstocks: Energy use in steam cracking and alternative processes. *Energy* **31**, 425–451 (2006).

5. H. G. Hao, Y. F. Zhao, D. M. Chen, J. M. Yu, K. Tan, S. Ma, Y. Chabal, Z. M. Zhang, J. M. Dou, Z. H. Xiao. Simultaneous trapping of C_2H_2 and C_2H_6 from a ternary mixture of $C_2H_2/C_2H_4/C_2H_6$ in a robust metal–Organic framework for the purification of C_2H_4. *Angew. Chem. Int. Ed.* **130**, 16299–16303 (2018).

6. F. Studt, F. Abild-Pedersen, T. Bligaard, R. Z. Sørensen, C. H. Christensen, J. K. Nørskov, Identification of non-precious metal alloy catalysts for selective hydrogenation of acetylene. *Science* **320**, 1320–1322 (2008).

7. A. Cadiau, K. Adil, P. Bhatt, Y. Belmabkhout, M. Eddaoudi, A metal-organic framework–based splitter for separating propylene from propane. *Science* **353**, 137–140 (2016).

8. H. Li, L. Li, R.-B. Lin, W. Zhou, Z. Zhang, S. Xiang, B. Chen, Porous metal-organic frameworks for gas storage and separation: Status and challenges. *EnergyChem* **1**. 100006 (2019).

9. Y. Yang, L. Li, R.-B. Lin, Y. Ye, Z. Yao, L. Yang, F. Xiang, S. Chen, Z. Zhang, S. Xiang, Ethylene/ethane separation in a stable hydrogen-bonded organic framework through a gating mechanism. *Nat. Chem.* **13**, 933–939 (2021).

10. O. M. Yaghi, M. J. Kalmutzki, C. S. Diercks, *Introduction to Reticular Chemistry: Metal-Organic Frameworks and Covalent Organic Frameworks* (Wiley, 2019).

11. O. M. Yaghi, M. O'Keeffe, N. W. Ockwig, H. K. Chae, M. Eddaoudi, J. Kim, Reticular synthesis and the design of new materials. *Nature* **423**, 705–714 (2003).

12. L. Yang, S. Qian, X. Wang, X. Cui, B. Chen, H. Xing, Energy-efficient separation alternatives: Metal–organic frameworks and membranes for hydrocarbon separation. *Chem. Soc. Rev.* **49**, 5359–5406 (2020).

13. R.-B. Lin, Z. Zhang, B. Chen, Achieving high performance metal-organic framework materials through pore engineering. *Acc. Chem. Res.* **54**, 3362–3376 (2021).

14. L. Yang, X. Cui, Q. Yang, S. Qian, H. Wu, Z. Bao, Z. Zhang, Q. Ren, W. Zhou, B. Chen, A single-molecule propyne trap: Highly efficient removal of propyne from propylene with anion-pillared ultramicroporous materials. *Adv. Mater.* **30**, 1705374 (2018).

15. L. Li, H. M. Wen, C. He, R. B. Lin, R. Krishna, H. Wu, W. Zhou, J. Li, B. Li, B. Chen, A metal-organic framework with suitable pore size and specific functional sites for the removal of trace propyne from propylene. *Angew. Chem. Int. Ed.* **130**, 15403–15408 (2018).

16. L. Yang, A. Jin, L. Ge, X. Cui, H. Xing, A novel interpenetrated anion-pillared porous material with high water tolerance afforded efficient C_2H_2/C_2H_4 separation. *Chem. Comm.* **55**, 5001–5004 (2019).

17. M. Jiang, X. Cui, L. Yang, Q. Yang, Z. Zhang, Y. Yang, H. Xing, A thermostable anion-pillared metal-organic framework for C_2H_2/C_2H_4 and C_2H_2/CO_2 separations. *Chem. Eng. J.* **352**, 803–810 (2018).

18. Z. Zhang, Q. Ding, X. Cui, X.-M. Jiang, H. Xing, Fine-tuning and selective-binding within an anion-functionalized ultramicroporous metal-organic framework for efficient olefin/paraffin separation. *ACS Appl. Mater. Interfaces* **12**, 40229–40235 (2020).

19. J.-R. Li, R. J. Kuppler, H.-C. Zhou, Selective gas adsorption and separation in metal-organic frameworks. *Chem. Soc. Rev.* **38**, 1477–1504 (2009).

20. B. Zhu, J.-W. Cao, S. Mukherjee, T. Pham, T. Zhang, T. Wang, X. Jiang, K. A. Forrest, M. J. Zaworotko, K.-J. Chen, Pore engineering for one-step ethylene purification from a three-component hydrocarbon mixture. *J. Am. Chem. Soc.* **143**, 1485–1492 (2021).

21. Z. Xu, X. Xiong, J. Xiong, R. Krishna, L. Li, Y. Fan, F. Luo, B. Chen, A robust Th-azole framework for highly efficient purification of C_2H_4 from a $C_2H_4/C_2H_2/C_2H_6$ mixture. *Nat. Commun.* **11**, 1–9 (2020).

22. Y. Wang, C. Hao, W. Fan, M. Fu, X. Wang, Z. Wang, L. Zhu, Y. Li, X. Lu, F. Dai, Z. Kang, R. Wang, W. Guo, S. Hu, D. Sun, One-step ethylene purification from an acetylene/ethylene/ethane ternary mixture by cyclopentadiene cobalt-functionalized metal–organic frameworks. *Angew. Chem. Int. Ed.* **60**, 11350–11358 (2021).

23. S.-Q. Yang, F.-Z. Sun, P. Liu, L. Li, R. Krishna, Y.-H. Zhang, Q. Li, L. Zhou, T.-L. Hu, Efficient purification of ethylene from C_2 hydrocarbons with an C_2H_6/C_2H_2-selective metal–organic framework. *ACS Appl. Mater. Interfaces* **13**, 962–969 (2020).

24. L. Fan, P. Zhou, X. Wang, L. Yue, L. Li, Y. He, Rational construction and performance regulation of an In(III)–tetraisophthalate framework for one-step adsorption-phase purification of C_2H_4 from C_2 hydrocarbons. *Inorg. Chem.* **60**, 10819–10829 (2021).

25. Z. Jiang, L. Fan, P. Zhou, T. Xu, S. Hu, J. Chen, D.-L. Chen, Y. He, An aromatic-rich cage-based MOF with inorganic chloride ions decorating the pore surface displaying the preferential adsorption of C_2H_2 and C_2H_6 over C_2H_4. *Inorg. Chem. Front.* **8**, 1243–1252 (2021).

26. R. E. Sikma, N. Katyal, S.-K. Lee, J. W. Fryer, C. G. Romero, S. K. Emslie, E. L. Taylor, V. M. Lynch, J.-S. Chang, G. Henkelman, Low-valent metal ions as MOF pillars: A new route toward stable and multifunctional MOFs. *J. Am. Chem. Soc.* **143**, 13710–13720 (2021).

27. P. Liu, Y. Wang, Y. Chen, J. Yang, X. Wang, L. Li, J. Li, Construction of saturated coordination titanium-based metal–organic framework for one-step $C_2H_2/C_2H_6/C_2H_4$ separation. *Sep. Purif. Technol.* **276**, 119284 (2021).

28. X. Cui, K. Chen, H. Xing, Q. Yang, R. Krishna, Z. Bao, H. Wu, W. Zhou, X. Dong, Y. Han, Pore chemistry and size control in hybrid porous materials for acetylene capture from ethylene. *Science* **353**, 141–144 (2016).

29. J. Wang, Y. Zhang, P. Zhang, J. Hu, R.-B. Lin, Q. Deng, Z. Zeng, H. Xing, S. Deng, B. Chen, Optimizing pore space for flexible-robust metal-organic framework to boost trace acetylene removal. *J. Am. Chem. Soc.* **142**, 9744–9751 (2020).

30. R.-B. Lin, L. Li, H. Wu, H. Arman, B. Li, R.-G. Lin, W. Zhou, B. Chen, Optimized separation of acetylene from carbon dioxide and ethylene in a microporous material. *J. Am. Chem. Soc.* **139**, 8022–8028 (2017).

31. J. Shen, X. He, T. Ke, R. Krishna, J. M. van Baten, R. Chen, Z. Bao, H. Xing, M. Dincă, Z. Zhang, Simultaneous interlayer and intralayer space control in two-dimensional metal-organic frameworks for acetylene/ethylene separation. *Nat. Commun.* **11**, 6259 (2020).

32. E. D. Bloch, W. L. Queen, R. Krishna, J. M. Zadrozny, C. M. Brown, J. R. Long, Hydrocarbon separations in a metal-organic framework with open iron (II) coordination sites. *Science* **335**, 1606–1610 (2012).

33. L. Li, R.-B. Lin, R. Krishna, H. Li, S. Xiang, H. Wu, J. Li, W. Zhou, B. Chen, Ethane/ethylene separation in a metal-organic framework with iron-peroxo sites. *Science* **362**, 443–446 (2018).

34. O. T. Qazvini, R. Babarao, Z.-L. Shi, Y.-B. Zhang, S. G. Telfer, A robust ethane-trapping metal-organic framework with a high capacity for ethylene purification. *J. Am. Chem. Soc.* **141**, 5014–5020 (2019).

35. R.-B. Lin, H. Wu, L. Li, X.-L. Tang, Z. Li, J. Gao, H. Cui, W. Zhou, B. Chen, Boosting ethane/ethylene separation within isoreticular ultramicroporous metal–organic frameworks. *J. Am. Chem. Soc.* **140**, 12940–12946 (2018).

36. S. Chen, Y. Li, Z. Bu, F. Yang, J. Luo, Q. An, Z. Zeng, J. Wang, S. Deng, Boosting CO_2-to-CO conversion on a robust single-atom copper decorated carbon catalyst by enhancing intermediate binding strength. *J. Mater. Chem. A* **9**, 1705–1712 (2021).

37. J. D. Yi, D. H. Si, R. Xie, Q. Yin, M. D. Zhang, Q. Wu, G. L. Chai, Y. B. Huang, R. Cao, Conductive two-dimensional phthalocyanine-based metal-organic framework nanosheets for efficient electroreduction of CO_2. *Angew. Chem. Int. Ed.* **133**, 17245–17251 (2021).

38. Q.-L. Qian, X.-W. Gu, J. Pei, H.-M. Wen, H. Wu, W. Zhou, B. Li, G. Qian, A novel anion-pillared metal–organic framework for highly efficient separation of acetylene from ethylene and carbon dioxide. *J. Mater. Chem. A* **9**, 9248–9255 (2021).

39. M. Thommes, K. Kaneko, A. V. Neimark, J. P. Olivier, F. Rodriguez-Reinoso, J. Rouquerol, K. S. Sing, Physisorption of gases, with special reference to the evaluation of surface area and pore size distribution (IUPAC Technical Report). *Pure Appl. Chem.* **87**, 1051–1069 (2015).

40. P. Zhang, Y. Zhong, J. Ding, J. Wang, M. Xu, Q. Deng, Z. Zeng, S. Deng, A new choice of polymer precursor for solvent-free method: Preparation of N-enriched porous carbons for highly selective CO_2 capture. *Chem. Eng. J.* **355**, 963–973 (2019).

41. P. Zhang, J. Wang, W. Fan, Y. Zhong, Y. Zhang, Q. Deng, Z. Zeng, S. Deng, Ultramicroporous carbons with extremely narrow pore size distribution via in-situ ionic activation for efficient gas-mixture separation. *Chem. Eng. J.* **375**, 121931 (2019).

42. P.-Q. Liao, W.-X. Zhang, J.-P. Zhang, X.-M. Chen, Efficient purification of ethene by an ethane-trapping metal-organic framework. *Nat. Commun.* **6**, 8697 (2015).

43. Y. X. Wang, S. Yuan, Z. G. Hu, T. Kundu, J. Zhang, S. B. Peh, Y. D. Cheng, J. Q. Dong, D. Q. Yuan, H. C. Zhou, D. Zhao, Pore size reduction in zirconium metal-organic frameworks for ethylene/ethane separation. *ACS Sustain. Chem. Eng.* **7**, 7118–7126 (2019).

44. C. Gucuyener, J. van den Bergh, J. Gascon, F. Kapteijn, Ethane/ethene separation turned on its head: Selective ethane adsorption on the metal-organic framework ZIF-7 through a gate-opening mechanism. *J. Am. Chem. Soc.* **132**, 17704–17706 (2010).

45. W. Yuan, X. Zhang, L. Li, Synthesis of zeolitic imidazolate framework-69 for adsorption separation of ethane and ethylene. *J. Solid State Chem.* **251**, 198–203 (2017).

46. L. Huang, D. P. Cao, Selective adsorption of olefin-paraffin on diamond-like frameworks: Diamondyne and PAF-302, *J. Mater. Chem. A* **1**, 9433–9439 (2013).

47. U. Böhme, B. Barth, C. Paula, A. Kuhnt, W. Schwieger, A. Mundstock, J. Caro, M. Hartmann, Ethene/ethane and propene/propane separation via the olefin and paraffin selective metal–organic framework adsorbents CPO-27 and ZIF-8. *Langmuir* **29**, 8592–8600 (2013).

48. D. Lv, R. Shi, Y. Chen, Y. Wu, H. Wu, H. Xi, Q. Xia, Z. Li, Selective adsorption of ethane over ethylene in PCN-245: Impacts of interpenetrated adsorbent. *ACS Appl. Mater. Interfaces***10**, 8366–8373 (2018).

49. Y. Chen, H. Wu, D. Lv, R. Shi, Y. Chen, Q. Xia, Z. Li, Highly adsorptive separation of ethane/ethylene by an ethane-selective MOF MIL-142A. *Ind. Eng. Chem. Res.* **57**, 4063–4069 (2018).

50. H. Wu, Y. Chen, D. Lv, R. Shi, Y. Chen, Z. Li, Q. Xia, An indium-based ethane-trapping MOF for efficient selective separation of C_2H_6/C_2H_4 mixture. *Sep. Purif. Technol.* **212**, 51–56 (2019).

51. J. Pires, M. L. Pinto, V. K. Saini, Ethane selective IRMOF-8 and its significance in ethane-ethylene separation by adsorption. *ACS Appl. Mater. Interfaces* **6**, 12093–12099 (2014).

52. H. Xiang, Y. Shao, A. Ameen, H. Chen, W. Yang, P. Gorgojo, F. Siperstein, X. Fan, Q. Pan, Adsorptive separation of C_2H_6/C_2H_4 on metal-organic frameworks (MOFs) with pillared-layer structures. *Sep. Purif. Technol.* **242**, 116819 (2020).

53. W. Liang, F. Xu, X. Zhou, J. Xiao, Q. Xia, Y. Li, Z. Li, Ethane selective adsorbent $Ni(bdc)(ted)_{0.5}$ with high uptake and its significance in adsorption separation of ethane and ethylene. *Chem. Eng. Sci.* **148**, 275–281 (2016).

54. Y. Chen, Z. Qiao, H. Wu, D. Lv, R. Shi, Q. Xia, J. Zhou, Z. Li, An ethane-trapping MOF PCN-250 for highly selective adsorption of ethane over ethylene. *Chem. Eng. Sci.* **175**, 110–117 (2018).

55. K.-J. Chen, D. G. Madden, S. Mukherjee, T. Pham, K. A. Forrest, A. Kumar, B. Space, J. Kong, Q.-Y. Zhang, M. J. Zaworotko. Synergistic sorbent separation for one-step ethylene purification from a four-component mixture. *Science* **366**, 241–246 (2019).

56. S. Mukherjee, N. Kumar, A. A. Bezrukov, K. Tan, T. Pham, K. A. Forrest, K. A. Oyekan, O. T. Qazvini, D. G. Madden, B. Space, M. J. Zaworotko, Amino-functionalised hybrid ultramicroporous materials that enable single-step ethylene purification from a ternary mixture. *Angew. Chem. Int. Ed.* **60**, 10902–10909 (2021).

57. B. C. Smith, in *Fundamentals of Fourier Transform Infrared Spectroscopy* (CRC Press, ed. 2, 2011), p. 207.

58. A. Dutta, Fourier transform infrared spectroscopy, in *Spectroscopic Methods for Nanomaterials Characterization* (Maharashtra Institute of Technology, 2017) pp. 73–93.
